# Radiotherapy for Locally Advanced Pancreatic Cancer in the Modern Era: A Systematic Review and Meta-Analysis

**DOI:** 10.3390/cancers17182959

**Published:** 2025-09-10

**Authors:** Sun Hyun Bae, Won Il Jang, Jeong Il Yu, Hee Chul Park, Ji Eun Moon, Karin Haustermans, Marta Scorsetti, Morten Høyer, Mi Sook Kim

**Affiliations:** 1 Department of Radiation Oncology, Soonchunhyang University College of Medicine, Bucheon 14584, Republic of Korea; gurigurihaia@hanmail.net; 2 Department of Radiation Oncology, Korea Institute of Radiological and Medical Sciences, Seoul 01812, Republic of Korea; 3 Department of Radiation Oncology, Samsung Medical Center, Sungkyunkwan University School of Medicine, Seoul 06351, Republic of Korea; ro.yuji651@gmail.com (J.I.Y.); hee.ro.park@samsung.com (H.C.P.); 4 Department of Biostatistics, Soonchunhyang University College of Medicine, Bucheon 14584, Republic of Korea; moon6188@schmc.ac.kr; 5 Department of Radiation Oncology, University Hospitals Leuven, 3000 Leuven, Belgium; karin.haustermans@uzleuven.be; 6 Radiotherapy and Radiosurgery Department, IRCCS Humanitas Research Hospital, Rozzano, 20089 Milan, Italy; marta.scorsetti@hunimed.eu; 7 Department of Biomedical Sciences, Humanitas University, Pieve Emanuele, 20072 Milan, Italy; 8 Danish Center for Particle Therapy, Aarhus University Hospital, 8200 Aarhus, Denmark

**Keywords:** intensity-modulated radiotherapy, modern radiotherapy, pancreatic cancer, particle beam therapy, stereotactic radiotherapy

## Abstract

**Background:** The optimal treatment strategy for locally advanced unresectable pancreatic cancer (LAPC) is still investigated. Therefore, we evaluated the role of radiotherapy (RT) in the management of LAPC in the modern era. **Methods:** A systematic review was conducted following the Preferred Reporting Items for Systemic Review and Meta-Analyses guidelines. Eligible studies were about for LAPC treated with curative-intent modern RT techniques including intensity-modulated radiotherapy (IMRT), stereotactic body radiotherapy (SBRT), and particle beam therapy (PBT) until September 2024. **Results:** In total, 53 observational studies, encompassing 2548 patients (993 treated with IMRT, 998 with SBRT, and 557 with PBT), met the inclusion criteria. Concurrent chemoradiotherapy (CCRT) was implemented in 28 studies, including only 3 studies in the SBRT group. Elective nodal irradiation (ENI) was adopted in 22%. The pooled 2-year overall survival (OS) rate was 29% (95% confidence interval [CI], 25–34%) for all patients, with no significant differences among RT techniques: 28% (95% CI, 22–34%) for IMRT, 26% (95% CI, 19–34%) for SBRT, and 43% (95% CI, 28–57%) for PBT (*p* = 0.1121). The pooled rate of acute hematologic toxicity (HT) ≥ grade 3 was 17% (95% CI, 9–26%), with significant differences among RT techniques: 23% (95% CI, 9–40%) for IMRT, 4% (95% CI, 0–11%) for SBRT, and 20% (95% CI, 6–37%) for PBT (*p* = 0.0181). In addition, CCRT (*p* = 0.0084) and ENI (*p* = 0.0145) significantly increased the risk of acute HT. Gastrointestinal toxicities rarely occurred. **Conclusions:** This systematic review and meta-analysis showed similar efficacy among modern RT techniques for LAPC management. Since almost all studies have single-arm design, and chemotherapy regimens have changed over time, conclusions must be drawn with caution. The use of modern RT techniques is individually selected according to clinical practice and resource availability.

## Simple Summary

The optimal treatment strategy for locally advanced unresectable pancreatic cancer (LAPC) is still investigated and the current standard treatment is clinical trial. The practice of radiotherapy (RT) is shifting worldwide from 3-dimensional conformal RT to modern RT techniques for LAPC, but there is insufficient evidence whether these advanced RT techniques would translate into better treatment outcomes. This is the first systematic review and meta-analysis focusing on modern RT techniques for LAPC. Modern RT techniques for LAPC show favorable survival outcomes and low toxicity rates, compared with historical data. Considering modern RT techniques showed similar efficacy, the optimal RT technique is individually selected according to clinical practice and resource availability.

## 1. Introduction

Pancreatic cancer is one of the most lethal malignancies globally, with a 5-year overall survival (OS) rates of <12% [1]. At initial presentation, approximately one-third of patients are diagnosed with locally advanced pancreatic cancer (LAPC) [2]. The current standard treatment for LAPC involves clinical trial or combination systemic chemotherapy [3,4,5]. However, there is no clear evidence that favors one regimen over another: regimens, such as nanoparticle albumin-bound paclitaxel + gemcitabine (AG) or oxaliplatin + irinotecan + fluorouracil + leucovorin (FOLFIRINOX), are derived on extrapolations from randomized trials in the metastatic setting. While a small subset of patients with excellent responses to chemotherapy may become eligible for surgical resection, most have incurable disease with high rates of local progression [6].

Theoretically, combining radiotherapy (RT) with chemotherapy could provide local disease control in addition to systemic disease control. However, several phase III randomized trials investigating concurrent chemoradiotherapy (CCRT) using two-dimensional (2D) or three-dimensional conformal RT (3DCRT) for LAPC have reported conflicting results regarding OS, despite demonstrating improved local control in patients treated with CCRT [7,8,9,10,11]. A meta-analysis from 2018 of five phase III trials for LAPC revealed that CCRT did not confer a survival benefit compared with chemotherapy alone and was associated with increased rates of grade 3 or 4 toxicities [12]. Consequently, CCRT using conventional fractionations is currently recommended as a treatment option only for selected patients with LAPC.

On the other hand, stereotactic body RT (SBRT), a novel RT technique delivering higher doses to the tumor in a few fractions, has been applied for LAPC and several prospective and retrospective studies have recently reported promising results [13,14]. Although SBRT is never evaluated in randomized trials, current international guidelines suggest SBRT as a viable treatment option for LAPC [15]. In addition, other new RT technologies, intensity-modulated RT (IMRT) and particle beam therapy (PBT), are increasingly being utilized and investigated for LAPC in clinical settings to improve the efficacy and reduce toxicity.

Globally, the practice of RT is transitioning from 3DCRT to modern RT techniques, including IMRT, SBRT, and PBT, across all cancers, but there is insufficient evidence whether these advanced RT techniques would translate into better treatment outcomes [16,17]. To address this gap, we conducted a comprehensive evaluation of the role of RT in LAPC management in the modern era. A systematic review and meta-analysis were performed to assess overall treatment outcomes of modern RT techniques for LAPC and compare the results of IMRT, SBRT, and PBT.

## 2. Methods and Materials

This systematic review adheres to the Preferred Reporting Items for Systematic Reviews and Meta-Analyses (PRISMA) guidelines (Supplementary Table S1) [18]. The study protocol was registered with the (CRD42024588555).

### 2.1. Search Strategy

The search strategy was developed by all authors in consultation with a professional librarian at Soonchunhyang University College of Medicine, Bucheon. Specific keywords were identified using the population, intervention, comparison, and outcome (PICO) model (Supplementary Table S2). A comprehensive literature search was conducted across databases, including MEDLINE (PubMed), EMBASE, Cochrane Library, and Web of Science through 19 September 2024. Only full-text articles on humans, published in English between 1979 and September 2024 were included. Additionally, relevant reviews and references were manually screened for any additional studies that were not identified in the original literature search. Two independent authors independently conducted the screening process to determine study eligibility. Disagreements were resolved by a senior author.

### 2.2. Selection Criteria

Only studies evaluating the treatment outcomes of modern RT techniques including IMRT, SBRT, and PBT to treat LAPC for radical intent were included. LAPC has several definitions, but we defined it as non-metastasized and unresectable pancreatic cancer. For this study, we defined SBRT as delivering ≥5 gray (Gy) per fraction in ≤6 fractions. Eligible studies were prospective or retrospective in design, included ≥10 patients, and reported at least one relevant endpoint, such as survivals and/or toxicities. When overlapping patient populations were present, the study with the largest sample size was selected. However, studies from the same institution were independently included if they were reported in distinct periods or with individual outcomes. The following were excluded: studies involving pediatric patients, studies evaluating patients with recurrent disease or distant metastases, and studies involving patients with a history of prior RT.

### 2.3. Data Extraction

Relevant data were independently extracted by two authors, focusing on patient demographics, treatment characteristics, survivals, and toxicities. The main outcomes focused on efficacy, including local progression-free survival (LPFS), PFS, OS, as well as safety, which included acute and late severe toxicities. Survival rates were obtained at 1-, 2-, and 3-year intervals after the start of initial treatment. In cases where data were presented only graphically, survival outcomes were estimated indirectly from descriptive plots. Severe toxicity was mainly defined by crude rates of grade ≥ 3 toxicity by the Common Terminology Criteria for Adverse Events (CTCAE) scale. Specific toxicity endpoints included acute hematologic toxicity (HT) and gastrointestinal toxicity (GIT) during RT, as well as late GIT following RT.

### 2.4. Quality Assessment

The quality assessment of non-randomized studies, including case-control and cohort studies, was performed using the Newcastle-Ottawa Scale (NOS) [19]. Each study was assigned a score ranging from 1 to 9: studies scoring between 7 and 9 points were classified as high-quality, those scoring 4 and 6 points were deemed medium quality, and studies scoring 1 and 3 points were regarded as low quality.

### 2.5. Statistical Analysis

Meta-analysis was conducted using the DerSimonian–Laird random effects model [20]. Considering the variation in RT techniques and chemotherapy regimens, changes in staging systems over time, and different study periods across the included studies, a random effects model was used over a fixed effects model. Heterogeneity was assessed by calculating Higgins’ (*I*^2^) statistic, where an *I*^2^ value > 50% indicated significant heterogeneity [21]. Publication bias was evaluated through visual inspection of Funnel plot symmetry. In addition, Egger’s regression test was performed to quantitatively assess the symmetry of the funnel plots. If the funnel plot was symmetrical or *p*-value was >0.05 in Egger’s test, then the null hypothesis of no publication bias was accepted. For subgroup comparisons, the Q test based on analysis of variance was used in conjunction with the random effects model. A *p* < 0.05 was considered statistically significant. Rex Excel-based statistical analysis software, version 3.6.3 (RexSoft, Seoul, Republic of Korea, https://rexsoft.org/) was used for the statistical analyses.

## 3. Results

**Figure 1:**
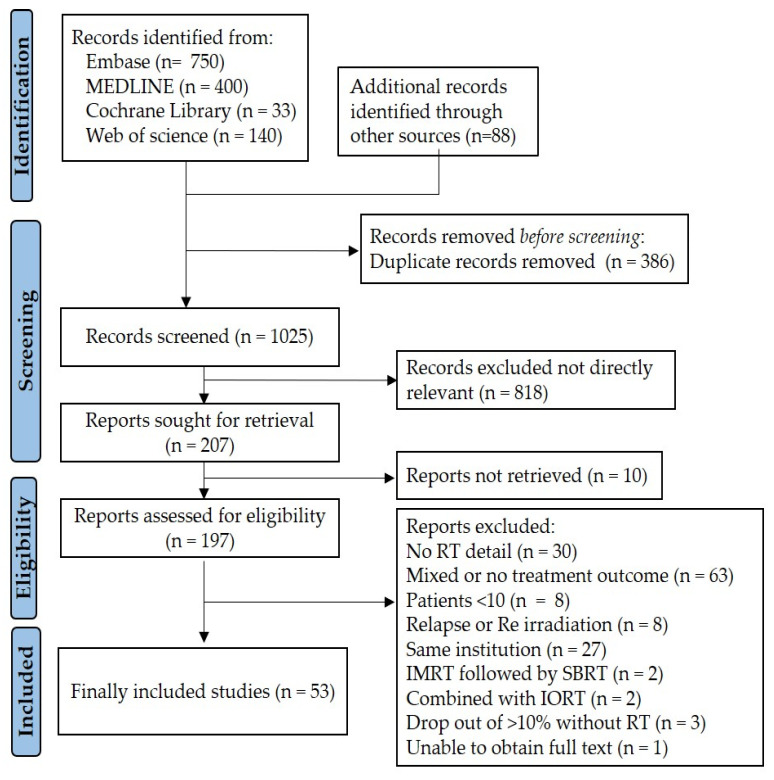
Flow diagram of study selection.

Initially, a total of 1411 studies, 1323 studies from four databases and 88 studies from additional sources, were identified. After multiple screening, 197 studies were selected for a full-text review and 144 were excluded for the following reason: (1) no treatment outcomes (n = 63); (2) no details on RT techniques (n = 30); (3) overlapping patients from same institution (n = 27); and so on. The PRISMA flow diagram, presented in Figure 1, outlines the detailed study selection process. Finally, 53 studies conducted between 2004 and 2024 met the inclusion criteria [22,23,24,25,26,27,28,29,30,31,32,33,34,35,36,37,38,39,40,41,42,43,44,45,46,47,48,49,50,51,52,53,54,55,56,57,58,59,60,61,62,63,64,65,66,67,68,69,70,71,72,73,74]. Among these, 2 studies separately analyzed the outcomes of patients with LAPC who received definitive RT into two treatment groups based on RT techniques, which were classified into distinct cohorts [23,33]. Ultimately, 55 cohorts compromising 2548 patients with unresectable LAPC were included for this study.

### 3.1. Study Characteristics

All 53 studies were prospective or retrospective observational studies. The quality of each study assessed using the NOS was medium to high (Table 1). Overall, 993 patients from 20 cohorts received IMRT, 998 patients from 25 cohorts received SBRT, and 557 patients from 10 cohorts received PBT. Induction chemotherapy (ICT) prior to RT was administered in 42 cohorts (76%): 18 cohorts pre-SBRT, 15 cohorts pre-IMRT, and 9 cohorts pre-PBT. The proportion of patients receiving ICT ranged from 0% to 100% across cohorts (median: 96%), with a median ICT duration of 1 month (range: 0–6 months). CCRT was employed in 28 cohorts (51%): 16 cohorts IMRT, 9 cohorts during PBT, and only 3 cohorts during SBRT. Since included studies were conducted during a long period of time, various chemotherapy regimens were utilized (Supplementary Table S3). The median RT dose was 24–75 Gy in 1–33 fractions (median 45 Gy in 10 fractions): 30–75 Gy in 10–33 fractions for IMRT, 24–45 Gy in 1–6 fractions for SBRT, and 30–67.5 Gy in 10–33 fractions for PBT (Supplementary Table S4). Since various fractionation schemes were used among studies, total doses were converted to the biologically equivalent dose (BED, GyE_10_) using a linear quadratic model with an α/β ratio of 10. The median BED_10_ was 37.5–112.5 Gy_10_ (median: 63.4 Gy_10_): 39–97.5 Gy_10_ (63.4 Gy_10_) for IMRT, 37.5–112.5 Gy_10_ (66.6 Gy_10_) for SBRT, and 39–85.7 Gy_10_ (75.5Gy_10_) for PBT. Elective nodal irradiation (ENI) was applied in 22% of cohorts.

### 3.2. Efficacy

Survival outcomes for individual cohorts following treatment initiation are summarized in Table 2. The median LPFS rates at 1-, 2-, and 3-year were 79% (range: 64–100%), 52% (20–90%), and 27% (12–90%), respectively. The median PFS rates at 1-, 2-, and 3-year were 43% (range: 0–72%), 14% (0–41%), and 11% (0–20%), respectively. The median OS ranged from 8 to 35 months, with a median of 16 months. The median OS rates at 1-, 2-, and 3-year were 71% (range: 24–95%), 29% (0–71%), and 13% (0–49%), respectively. According to RT techniques, the median OS rates at 1-, 2-, and 3-year were 67%, 31%, and 12% for IMRT; 69%, 25%, and 13% for SBRT; and 80%, 35%, and 16% for PBT, respectively.

**Figure 2:**
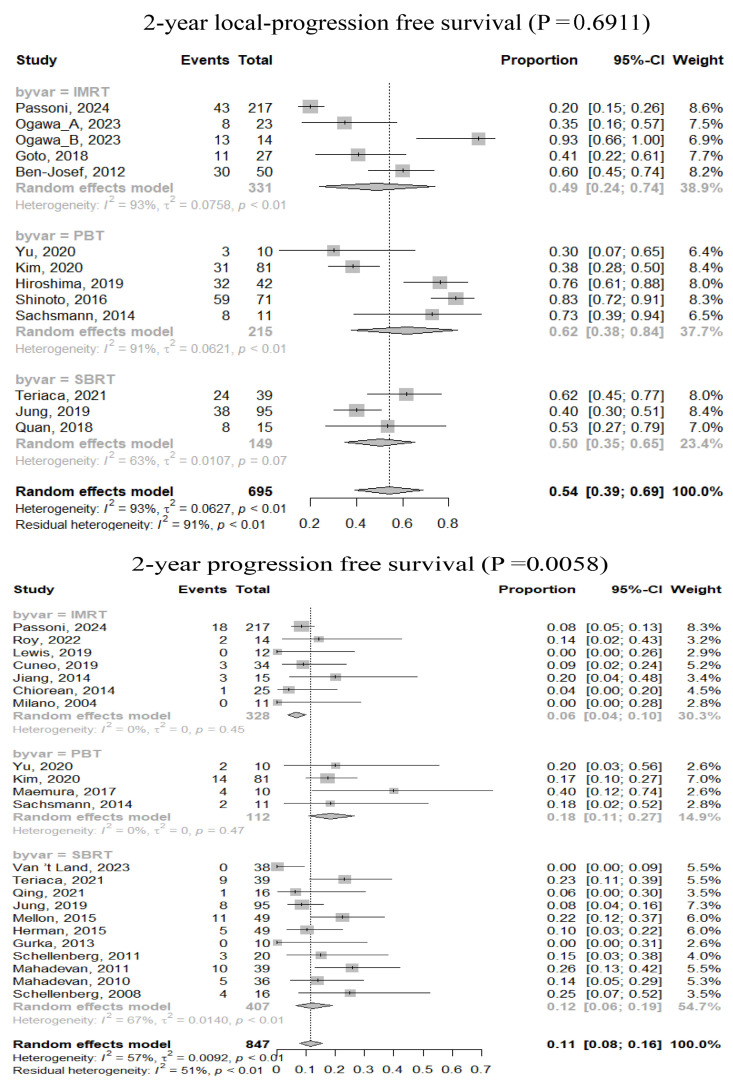

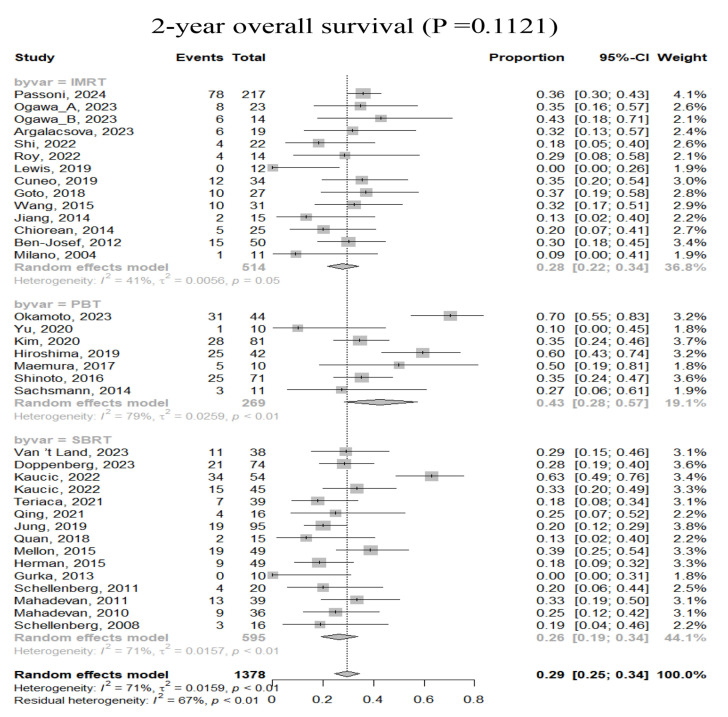
Forrest plots of survivals according to modern radiotherapy techniques [22,23,24,25,26,29,30,32,35,36,37,38,40,41,44,47,48,50,51,53,54,57,58,59,60,61,63,64,65,68,69,70,72,73,74].

Using random effects analysis, pooled rates for 2-year LPFS, PFS, and OS were 54% (95% confidence interval [CI], 39–69%), 11% (95% CI, 8–16%), and 29% (95% CI, 25–34%), respectively (Figure 2). Subgroup comparisons revealed no significant differences in treatment outcomes among RT techniques (Table 3, Supplementary Table S5). The duration of ICT was the only significant factor affecting OS: a median ICT duration of >1 month was associated with improved 1- and 2-year OS rates. Additionally, delivering a higher RT dose (mBED > 60 Gy_10_) was significantly associated with improved 3-year OS rate.

### 3.3. Safety

**Figure 3:**
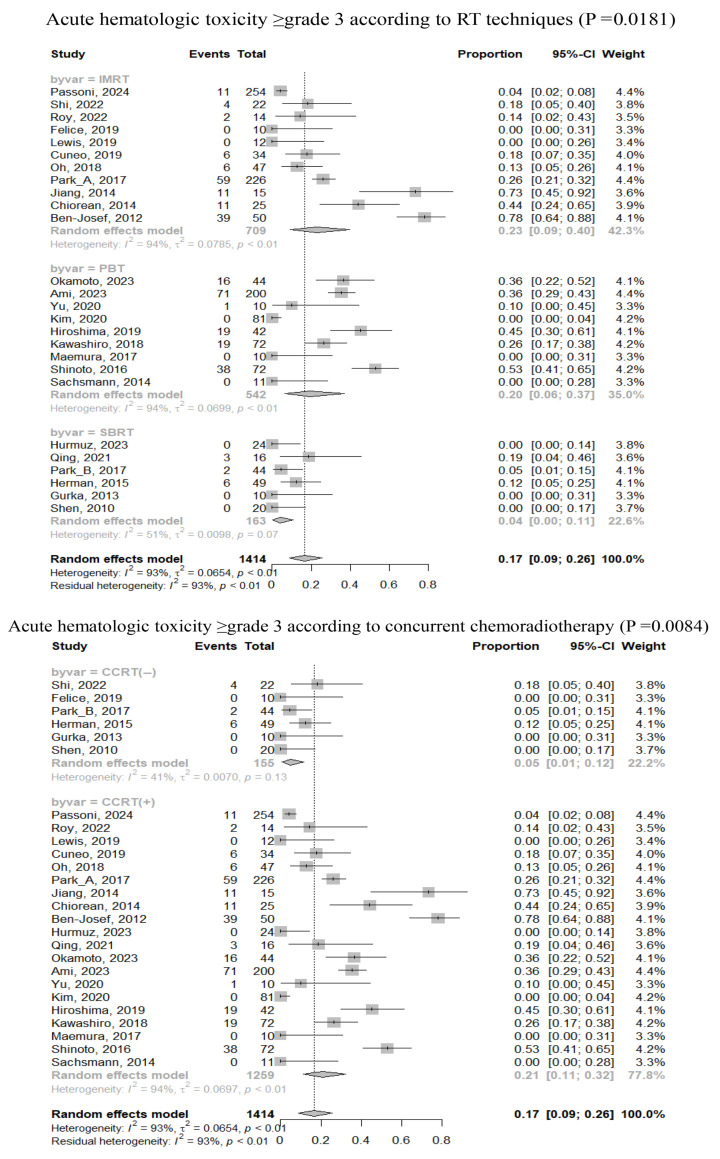

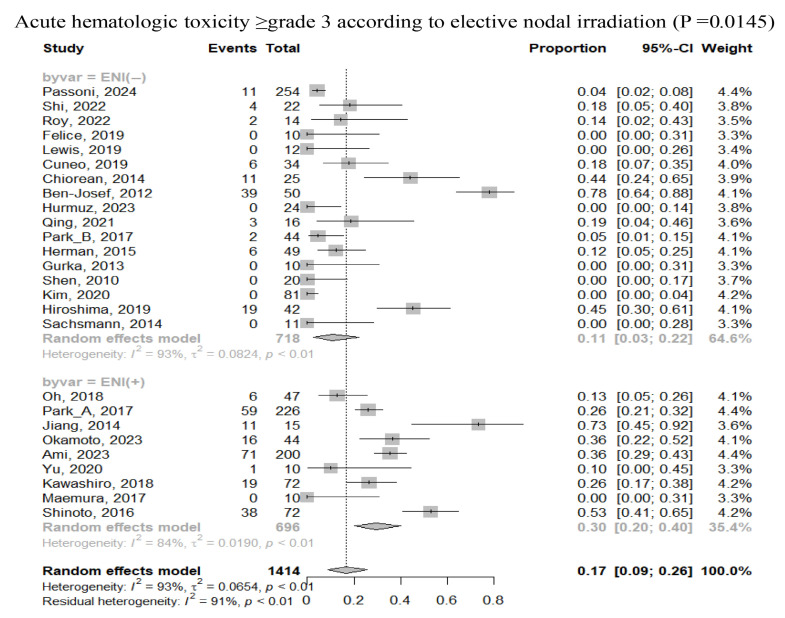
Forrest plots of acute severe toxicities ≥ grade 3 according to RT techniques, concurrent chemoradiotherapy, and elective nodal irradiation [22,25,26,28,29,30,31,33,36,37,38,43,51,58,59,62,65,67,68,69,70,71,72,73,74].

Details regarding the overall incidence of severe toxicities during and after RT are presented in Supplementary Table S6. Acute HT rates ≥ grade 3 during RT ranged from 0 to 78% of the patients. Acute and late GIT rates ≥ grade 3 were observed in 0–14% and 0–50% of patients, respectively. The pooled rate of acute HT ≥ grade 3 was 17% (95% CI, 9–26%), respectively. Subgroup analyses indicated that RT techniques, CCRT, and ENI were significant factors associated with acute HT (Figure 3). The pooled rates of acute and late GIT ≥ grade 3 were 0% (95% CI, 0–1%) and 2% (95% CI, 1–4%), respectively.

### 3.4. Publication Bias

Funnel plots and of Egger’s regression test results for the included studies are presented in Supplementary Figure S1. Significant publication bias was observed for 1-year OS outcome. Other than that, no publication bias was noted for other survival outcomes and toxicities.

## 4. Discussion

The role of RT in treating LAPC is still under debate. Mixed results were reported in several randomized trials comparing CCRT with chemotherapy alone for the treatment of LAPC [7,8,9,10,11]. Subsequent systematic review and meta-analysis including these phase III trials showed no significant difference in OS in patients treated with CCRT and chemotherapy alone (54% vs. 55% at 1-year; 15% vs. 14% at 2-year) [12]. These studies applied all 2D or 3DCRT. Anatomically, the pancreas is adjacent to radiosensitive organs at risk (OARs), especially the stomach and duodenum. The use of 2D or 3DCRT for LAPC limits the dose escalation to the tumor and carries the risk of GIT. On the other hand, in modern RT techniques, such as IMRT and SBRT, the ability to spare adjacent OARs while delivering a therapeutic dose to the target has improved [75]. One meta-analysis comparing 3DCRT with IMRT in PC revealed significant reductions in acute and late toxicities ≥ grade 3 with IMRT, despite similar survival outcomes [76]. Another meta-analysis reported both improved OS and reduced GIT ≥ grade 3 with IMRT compared to 3DCRT [77]. Reflecting this evolution in clinical practice, data from National Cancer Data base (NCDB) for patients with LAPC diagnosed between 2004 and 2019 showed a decline in 3DCRT usage from 66% in 2004 to 9% in 2019, alongside increases in IMRT from 30% to 62% and in SBRT from 4% to 29% [78].

In this study, pooled 1- and 2-year OS rates with modern RT techniques were 71% (95% CI, 66–76%), and 29% (95% CI, 25–34%), respectively. The pooled rates of acute and late GIT ≥ grade 3 were minimal at 0% (95% CI, 0–1%) and 2% (95% CI, 1–4%), respectively. To the best of our knowledge, this is the first systematic review and meta-analysis focusing on the efficacy and safety of modern RT techniques for unresectable LAPC, aligned with recent changes in clinical practice.

In spite of improved systemic control, the local control rate of conventional fractionated RT was insufficient, leading clinicians to change their preference from conventional fractionated CCRT to new RT techniques, i.e., SBRT [79]. Early SBRT studies using 1–3 fractions showed promising outcomes but were associated with unacceptable high rates of severe GIT [64,80,81]. A landmark phase II study using 33 Gy in five fractions following three cycles of gemcitabine reported a median OS of 13.9 months, with acute and late GIT rates ≥ grade 2 of 2% and 11%, respectively [58]. Since then, SBRT regimens using ≥5 fractions with 33–50 Gy are recommended [3,82].

Several studies were conducted to identify the optimal RT scheme for treating LAPC. One meta-analysis, comparing SBRT with conventional fractionated RT using 3DCRT or IMRT from studies published between 2004 and 2016, reported a significant OS benefit for the SBRT group [83]. NCDB for patients with LAPC diagnosed between 2004 and 2012 also showed a significant benefit for OS in the SBRT group compared to the IMRT group [84]. On the other hand, recent observational studies reported similar OS [15,33]. Consistent with these findings, the current meta-analysis, which included studies published between 2004 and 2024, showed similar efficacy between SBRT and IMRT. Ongoing phase II randomized trials comparing SBRT with IMRT could be an answer (NCT03704662).

Another tool to improve efficacy is PBT. Pancreatic cancer is considered to be a radioresistant tumor [85]. Potentially, the higher linear energy transfer and higher relative biological effectiveness of protons and heavy ions could lead to improved disease control in radioresistant tumor types [86,87]. In addition, PBT is characterized by its unique physical property, consisting of a finite range of dose deposit in tissues and no exit dose beyond the end of their path, the so-called Bragg peak [88]. Although clinical data on PBT for LAPC remains limited, available studies are very encouraging. Okamoto et al. [65] reported the highest median OS of 35 months from treatment initiation and 30 months following CCRT with carbon-ion RT. The present meta-analysis showed similar efficacy between PBT and photon-based RT using IMRT or SBRT. Additional studies are needed to determine the role of PBT for LAPC, considering limited clinical data on PBT presented on this study.

Traditional treatment strategy for LAPC is CCRT administered over 5–6 weeks, followed by chemotherapy. On the other hand, SBRT is completed quickly within 1–5 fractions, minimizing delays in chemotherapy initiation, and is generally well-tolerated by patients. Several SBRT studies tried new approaches, such as upfront SBRT followed by chemotherapy; however, early distant metastases occurred soon after SBRT in some patients [56,63].

As approximately 30% of patients with LAPC present occult metastatic disease at diagnosis, ICT can help to select a subgroup of patients without early metastatic progression who can potentially better benefit from RT [89]. Based on this, a new treatment strategy, composing of ICT followed by CCRT or SBRT, has emerged. Two retrospective studies reported that ICT followed by CCRT had statistically superior OS compared to chemotherapy alone or upfront CCRT [90,91]. Similarly, NCDB study reported the best benefit in patients with LAPC treated with ICT followed by CCRT [92]. The LAP-07 phase III trial showed that CCRT after 4 months of ICT delayed locoregional progression, although it did not translate into survival benefit [11]. Our meta-analysis identified a median ICT duration > 1 month as a significant factor influencing OS. American Society of Clinical Oncology and National Comprehensive Cancer Network guidelines recommend 4–6 months or 4–6 cycles of ICT followed by CCRT or SBRT for selected patients without systemic progression [3,5]. On the other hand, 2023 European Society for Medical Oncology guidelines recommend the same treatment strategy but do not specify the ICT duration [4]. Clinically preferred regimens for LAPC are AG or FOLFIRINOX, but the efficacy and safety of combining these regimens with RT remain under investigation. One prospective cohort study reported that 30% of patients with LAPC could not complete first-line chemotherapy, with 57% discontinuing due to disease progression and 43% due to toxicities [93]. In particular, grade 3–4 HT commonly occur with the use of AG or FOLFIRINOX [94]. Our meta-analysis revealed that CCRT was the most significant factor for HT ≥grade 3, whereas HT rarely occurred in patients undergoing SBRT. Considering that only three studies of SBRT applied CCRT with less toxic oral agents (capecitabine or S-1), or experimental vaccine, we think bone marrow suppression caused by concurrent cytotoxic agents may play a more significant role in HT than bone marrow-sparing properties by IMRT or PBT. Multiple ongoing prospective studies using various combinations of modern RT techniques and modern chemotherapy regimens for LAPC would give some evidence to better determine the optimal treatment strategy [95].

Technical advance from 2DRT to 3DCRT requires the change in practices from using solely bony and muscular landmarks to a more complex use of computed tomography-based anatomy to define target volume of the tumor and regional lymph nodes (LN), and results in wide variation in the target delineation across all cancers [96]. Consequently, the development of standardized consensus and contouring guidelines is crucial to minimizing variability. In pancreatic cancer, target delineation remains controversial, especially regarding ENI. In adjuvant setting, regional LNs around a resected tumor is a major failure site and the inclusion of ENI according to Radiation Therapy Oncology Group consensus panel guidelines is standard practice since 2012 [97]. On the other hand, it is relatively rare in LAPC, especially when chemotherapy regimens such as gemcitabine or FOLFIRINOX are combined. These may be effective in controlling micrometastases in regional LNs. In addition, the inclusion of ENI increases the target volume and risk of GIT located near the pancreas. Murphy et al. [98] treated only gross tumor volume plus a 1 cm margin with 36 Gy in 15 fractions for 74 patients with LAPC. They omitted ENI but only 4 patients (5%) experienced regional LN failure: 3 in-field and 1 marginal failure. 16 patients (22%) had GIT ≥grade 3 and planning target volume significantly correlated with GIT (*p* = 0.0070). A committee of European Society for Radiotherapy and Oncology do not recommend ENI patients receive CCRT or SBRT for LAPC [99]. Practice guidelines published by the American Society for Radiation Oncology conditionally recommend ENI for CCRT but not routinely for SBRT [100]. In this meta-analysis, 12 cohorts (22%) included ENI, which significantly increased acute HT ≥ grade 3 during RT, although acute and late severe GIT were rare. Although there was no statistical difference for OS, a trend toward improved 3-year OS was observed in ENI (+) than ENI (−) (23% [95% CI, 11–37%] vs. 12% [95% CI, 8–16%], *p* = 0.0670). The present meta-analysis does not support ENI but suggests that subgroups potentially may have benefit from ENI.

The current meta-analysis has some limitations. First, the majority of included studies were retrospective, and no phase III randomized trials compared IMRT, SBRT, and PBT. We indirectly compared modern RT techniques from single-arm studies. The heterogeneity of observational studies and potential selection bias may have affected the pooled analysis [101]. Second, the meta-analysis encompassed 53 studies published between 2004 and 2024. During that time, there have been some improvements in chemotherapy and especially two regimens, FOLFIRINOX and AG, are recommended first for LAPC [102,103]. Although most patients in this analysis received chemotherapy, less than half received these modern regimens, which may have affected treatment outcomes (Supplementary Table S3). Regarding the best chemotherapy regimen, various regimens are recommended; however, our meta-analysis represents real clinical practice. Third, our results may be biased because the included studies used different definitions for LAPC. Further studies about the consensus on the definition of LAPC will be needed to select the optimal patients and compare clinical studies. Fourth, magnetic resonance (MR)-Linac is a novel RT machine that uses MR imaging acquired both before and continuously during treatment delivery and conducts on-table RT plan modification. These unique physics distinguish MR-Linac from other photon RT and are expected as a remedy for LAPC. However, MR-Linac operates in a limited number of facilities worldwide [104]. We found only two studies met our inclusion criteria (Supplementary Table S4) and we cannot analyze the advantage of MR-Linac, accordingly [44,46]. Lastly, we found several significant factors affecting OS in the subgroup analysis; however, we could not draw specific values to define the subgroup that benefited from modern RT techniques for LAPC in the absence of individual patient data.

## 5. Conclusions

From the current systematic review and meta-analysis, modern RT techniques for LAPC show favorable survival outcomes and low toxicity rates, compared with historical data. Between modern RT techniques, IMRT, SBRT, and PBT showed similar efficacy. Since almost all studies have single-arm design, and chemotherapy regimens have changed over time, conclusions must be drawn with caution. Therefore, the use of modern RT techniques is individually selected according to clinical practice and resource availability. Further studies will be needed to better evaluate the optimal RT scheme and find the combination strategy with the modern chemotherapy regimens.

## Figures and Tables

**Table 1:** Study characteristics.

Author	Year	Study	No. of pts	ICT (%)	CC RT (%)	ACT (%)	RT	mRT Dose (Gy)	mNo. of fx (Range)	ENI	NOS
Passoni [22]	2024	P/S	217	100	93	0	IMRT	44.3	15	No	5
Ogawa_A [23]	2023	R/S	23	100	100		IMRT	48	15	No	5
Ogawa_B [23]	2023	R/S	14	100	100		IMRT	48	15	No	5
Argalacsova [24]	2023	R/M	19	100	0		IMRT	39.9	15	No	8
Shi [25]	2022	R/S	22	100	0		IMRT	SIB: 50/30	10	No	5
Roy [26]	2022	P/M	14	0	100	0	IMRT	50.4	28	No	6
Reyngold [27]	2021	P/S	119	98	93	16	IMRT	75	25 (15 or 25)	Yes	5
Felice [28]	2019	P/S	10	80	0	0	IMRT	52/13	13	No	4
Lewis [29]	2019	P/S	12	0	100		IMRT	SIB: 57/45	25	No	5
Cuneo [30]	2019	P/S	34	100	100	100	IMRT	52.5	25	No	5
Oh [31]	2018	R/S	47	79	100	49	IMRT	SIB: 55/44	22	Yes	7
Goto [32]	2018	R/S	27	100	100		IMRT	48	15	Yes	7
Park_A [33]	2017	R/S	226	100	97		IMRT		NR (25–28)	Yes	8
Colbert [34]	2017	R/S	59	100	100		IMRT	63	28 (15–28)	No	5
Wang [35]	2015	R/S	31				IMRT	46	23	No	5
Jiang [36]	2014	P/S	15	0	100		IMRT	50.4	28	Yes	5
Chiorean [37]	2014	P/S	25	100	100	100	IMRT	SIB: 50/45	25	No	5
Ben-Josef [38]	2012	P/M	50	100	100	84	IMRT	55	25 (24–25)	No	5
Abelson [39]	2012	R/S	18	28	100	17	IMRT	54	30 (22–33)	Yes	6
Milano [40]	2004	R/S	11		100		IMRT	59.4	33 (28–33)	No	4
Van ’t Land [41]	2023	P/S	38	100	100	100	SBRT	40	5	No	5
Reyngold [42]	2023	P/M	24	100	0	0	SBRT	30	3	No	5
Hurmuz [43]	2023	R/S	24	67	34	67	SBRT	35	5 (3–5)	No	5
Doppenberg [44]	2023	R/S	74	100	0	30	SBRT	40	5 (4–5)	No	5
Comito [45]	2023	R/S	142	54	0	30	SBRT	45	6	No	5
Lee [46]	2022	R/S	33	100	0		SBRT		5	No	5
Kaucic [47]	2022	R/S	54		0		SBRT	45	3 (1–5)	No	5
Kaucic [48]	2022	R/S	45		0		SBRT	40	5 (3–5)	No	5
Zhu [49]	2021	P/S	63	0	0	100	SBRT	36	5	No	5
Teriaca [50]	2021	P/M	39	100	0	0	SBRT	40	5	No	6
Qing [51]	2021	P/S	16	0	100	87	SBRT	40	5	No	5
Bouchart [52]	2021	P/M	16	100	0		SBRT		5	No	5
Jung [53]	2019	R/S	95	14	0	81	SBRT	28	4 (4–5)	No	5
Quan [54]	2018	P/S	15	100	0	47	SBRT	36	3	No	5
Jumeau [55]	2018	R/S	17	29	0		SBRT	30	5 (5–6)	No	4
Heerkens [56]	2018	P/S	20	0	0	0	SBRT	24	3	No	5
Park_B [33]	2017	R/S	44	95	0		SBRT		5	No	8
Mellon [57]	2015	R/S	49	100	0		SBRT	SIB: 40/30	5	No	5
Herman [58]	2015	P/M	49	90	0	100	SBRT	33	5	No	6
Gurka [59]	2013	P/S	10	100	0	100	SBRT	25	5	No	5
Schellenberg [60]	2011	P/S	20	100	0	100	SBRT	25	1	No	5
Mahadevan [61]	2011	R/S	39	100	0	95	SBRT	24	3	No	5
Shen [62]	2010	R/S	20		0		SBRT	45	4 (3–6)	No	4
Mahadevan [63]	2010	R/S	36	0	0	86	SBRT	30	3	No	5
Schellenberg [64]	2008	P/S	16	100	0		SBRT	25	1	No	5
Okamoto [65]	2023	R/S	44	84	100	100	PBT	55.2	12	Yes	5
Lautenschlaeger [66]	2023	R/S	15	73			PBT			No	4
Ami [67]	2023	R/S	200	53	93		PBT	67.5	25	Yes	5
Yu [68]	2020	P/S	10	90	60		PBT	65.4	33 (32–34)	Yes	5
Kim [69]	2020	R/S	81	24	90	74	PBT	SIB: 45/30	10	No	7
Hiroshima [70]	2019	R/S	42	76	100	81	PBT	60	25 (25–33)	No	5
Kawashiro [71]	2018	R/M	72	74	78	0	PBT	55.2	12	Yes	6
Maemura [72]	2017	R/S	10	100	100	100	PBT	50	25	Yes	7
Shinoto [73]	2016	P/S	72	0	99	0	PBT		12	Yes	5
Sachsmann [74]	2014	P/S	11	73	100		PBT	59.4	33	No	5

Abbreviations: No.—number; ICT—induction chemotherapy before radiotherapy (RT); CCRT—concurrent chemoradiotherapy; ACT—adjuvant chemotherapy after RT; mRT dose—median RT dose; mNo.—median number; fx—fraction; ENI—elective nodal irradiation; NOS—the Newcastle-Ottawa Scale; P—prospective study; R—retrospective study; S—single center; M—multicenter; IMRT—intensity-modulated radiotherapy; SBRT—stereotactic body radiotherapy; PBT—particle beam therapy; SIB—simultaneous integral boost.

**Table 2:** Survival outcomes after initial treatment for unresectable, locally advanced pancreatic cancer.

Author	RT	1-yr LPFS (%)	2-yr LPFS (%)	3-yr LPFS (%)	1-yr PFS (%)	2-yr PFS (%)	3-yr PFS (%)	mOS (mo)	1-yr OS (%)	2-yr OS (%)	3-yr OS (%)
Passoni [22]	IMRT	71	20	12	51	8	3	20	85	36	13
Ogawa_A [23]	IMRT	73	33	25				18	78	35	10
Ogawa_B [23]	IMRT	90	90	90				17	65	43	37
Argalacsova [24]	IMRT							14	58	32	5
Shi [25]	IMRT				52			16	73	16	
Roy [26]	IMRT				35	18	18	11	50	27	18
Reyngold [27]	IMRT							27			
Felice [28]	IMRT				69				83		
Lewis [29]	IMRT				42	0	0	12	50	0	0
Cuneo [30]	IMRT			68	22	10	10	22	72	34	19
Oh [31]	IMRT							14			
Goto [32]	IMRT	73	43	22					92	38	28
Park_A [33]	IMRT										
Colbert [34]	IMRT										
Wang [35]	IMRT							16	62	32	9
Jiang [36]	IMRT				29	19	19	13	68	11	11
Chiorean [37]	IMRT				34	4	0	13	52	20	16
Ben-Josef [38]	IMRT	87	59	59				15	74	30	9
Abelson [39]	IMRT	64			16			8	24		
Milano [40]	IMRT				0	0		13	58	12	
Van ’t Land [41]	SBRT				47	0		19	82	29	
Reyngold [42]	SBRT										
Hurmuz [43]	SBRT										
Doppenberg [44]	SBRT							20	79	29	13
Comito [45]	SBRT										
Lee [46]	SBRT										
Kaucic [47]	SBRT	100			72			24	91	63	40
Kaucic [48]	SBRT	96			69			17	71	33	18
Zhu [49]	SBRT				12			14	73		
Teriaca [50]	SBRT	81	62	53	43	23	15	18	77	18	13
Qing [51]	SBRT	69			38	6		15	69	25	19
Bouchart [52]	SBRT							25	88		
Jung [53]	SBRT	80	40	15	43	8	3	17	67	20	5
Quan [54]	SBRT	78	52					14	60	16	
Jumeau [55]	SBRT							22			
Heerkens [56]	SBRT							9			
Park_B [33]	SBRT										
Mellon [57]	SBRT	78			58	22	15	15	78	38	9
Herman [58]	SBRT	78			32	10		14	59	18	
Gurka [59]	SBRT				20	0		12	50	0	
Schellenberg [60]	SBRT				37	17		12	50	20	7
Mahadevan [61]	SBRT				55	25		20	69	33	
Shen [62]	SBRT										
Mahadevan [63]	SBRT				30	14		14	51	25	
Schellenberg [64]	SBRT				51	25		11	50	18	
Okamoto [65]	PBT							35	95	71	49
Lautenschlaeger [66]	PBT										
Ami [67]	PBT										
Yu [68]	PBT	67	27	27	60	20	20	17	80	13	13
Kim [69]	PBT	79	38	24	45	18	11	19	73	35	7
Hiroshima [70]	PBT	90	77					28	85	59	
Kawashiro [71]	PBT										
Maemura [72]	PBT				60	41		22	80	45	23
Shinoto [73]	PBT	92	83	78				20	73	35	16
Sachsmann [74]	PBT	86	69		55	14		18	61	31	

Abbreviations: RT—radiotherapy; IMRT—intensity-modulated radiotherapy; SBRT—stereotactic body radiotherapy; PBT—particle beam therapy; LPFS—local progression-free survival; PFS—progression-free survival; mOS—median overall survival.

**Table 3:** Pooled rates of overall survival (OS) for unresectable, locally advanced pancreatic cancer treated with radiotherapy.

Group	Cohorts	N	*p*, Heterogeneity	*I* ^2^	Random Event Rate (95% CI)	*p* (Between Groups)
1-year OS	40	1485	<0.0001	69.29%	0.71 (0.66–0.76)	
IMRT	16	542	<0.0001	76.64%	0.67 (0.57–0.76)	0.1111
PBT	7	269	0.0175	60.99%	0.80 (0.71–0.89)	
SBRT	17	674	0.0005	61.55%	0.71 (0.65–0.77)	
mBED ≤ 60 Gy_10_	16	724	<0.0001	71.13%	0.66 (0.58–0.73)	0.1317
mBED > 60 Gy_10_	22	674	<0.0001	70.22%	0.74 (0.67–0.80)	
mICT > 1 mo	9	523	0.0025	66.41%	0.80 (0.72–0.86)	0.0031
mICT ≤ 1 mo	14	424	0.0040	57.39%	0.64 (0.56–0.72)	
ENI (−)	33	1290	<0.0001	61.58%	0.70 (0.66–0.75)	0.6109
ENI (+)	7	195	<0.0001	85.61%	0.76 (0.56–0.91)	
2-year OS	36	1378	<0.0001	70.57%	0.29 (0.25–0.34)	
IMRT	14	514	0.0522	41.46%	0.28 (0.22–0.34)	0.1121
PBT	7	269	<0.0001	78.73%	0.43 (0.28–0.57)	
SBRT	15	595	<0.0001	71.09%	0.26 (0.19–0.34)	
mBED ≤ 60 Gy_10_	16	724	0.0009	60.49%	0.25 (0.19–0.31)	0.1363
mBED > 60 Gy_10_	19	583	<0.0001	75.07%	0.33 (0.25–0.41)	
mICT > 1 mo	8	507	<0.0001	78.13%	0.34 (0.25–0.45)	0.0439
mICT ≤ 1 mo	13	361	0.0350	46.03%	0.22 (0.16–0.28)	
ENI (−)	30	1201	<0.0001	65.75%	0.28 (0.23–0.33)	0.3641
ENI (+)	6	177	<0.0001	81.20%	0.37 (0.19–0.56)	
3-year OS	25	1089	<0.0001	68.51%	0.14 (0.10–0.19)	
IMRT	12	481	0.1987	24.95%	0.13 (0.09–0.17)	0.7400
PBT	5	216	<0.0001	85.16%	0.19 (0.05–0.37)	
SBRT	8	392	<0.0001	78.37%	0.14 (0.07–0.23)	
mBED ≤ 60 Gy_10_	11	568	0.4707	0%	0.09 (0.06–0.11)	0.0040
mBED > 60 Gy_10_	13	450	<0.0001	72.58%	0.20 (0.12–0.28)	
mICT > 1 mo	5	415	<0.0001	86.35%	0.18 (0.08–0.30)	0.5665
mICT ≤ 1 mo	9	250	0.2772	18.62%	0.13 (0.08–0.19)	
ENI (−)	19	912	0.0005	59.73%	0.12 (0.08–0.16)	0.0670
ENI (+)	6	177	0.0066	68.91%	0.23 (0.11–0.37)	

Abbreviations: N—number of patients; IMRT—intensity-modulated radiotherapy; SBRT—stereotactic body radiotherapy; PBT—particle beam therapy; mBED—median biologically equivalent dose, which was calculated using an α/β ratio of 10; mICT—median duration of induction chemotherapy before RT; ENI—elective nodal ir.

## Data Availability

All data generated or analyzed during this study are included in this published article.

## Supplementary Materials

The following supporting information can be downloaded at: https://www.mdpi.com/article/10.3390/cancers17182959/s1, Figure S1: Funnel plots and *p*-value of Egger’s regression tests; Table S1: PRISMA 2020 Checklist; Table S2: Search strategy and results; Table S3: Study details on chemotherapy; Table S4: Study details on radiotherapy; Table S5: Pooled rates of local progression-free survival and progression-free survival; Table S6: Severe toxicities ≥grade 3.
