# Supplementary material for: Radiotherapy for Locally Advanced Pancreatic Cancer in the Modern Era: A Systematic Review and Meta-Analysis
Source: Cancers (Basel). 2025 Sep 10;17(18):2959. doi: 10.3390/cancers17182959 (PMC12468599; doi:10.3390/cancers17182959)
Supplement: Supplementary file 1 [file cancers-17-02959-s001.zip › cancers-3813738-supplementary.pdf]

SUPPLIMEMTARY CONTENT

Suppl. Figure. 1. Funnel plots and *P*-value of Egger's regression tests

Suppl. Table 1. PRISMA 2020 Checklist

Suppl. Table 2. Search strategy and results

Suppl. Table 3. Study details on chemotherapy

Suppl. Table 4. Study details on radiotherapy

Suppl. Table 5. Pooled rates of local progression-free survival and progression-free survival

Suppl. Table 6. Severe toxicities ≥grade 3

Supplementary Figure. 1. Funnel plots and *P*-value of Egger's regression tests

Funnel plot of 1-year local progression-free survival

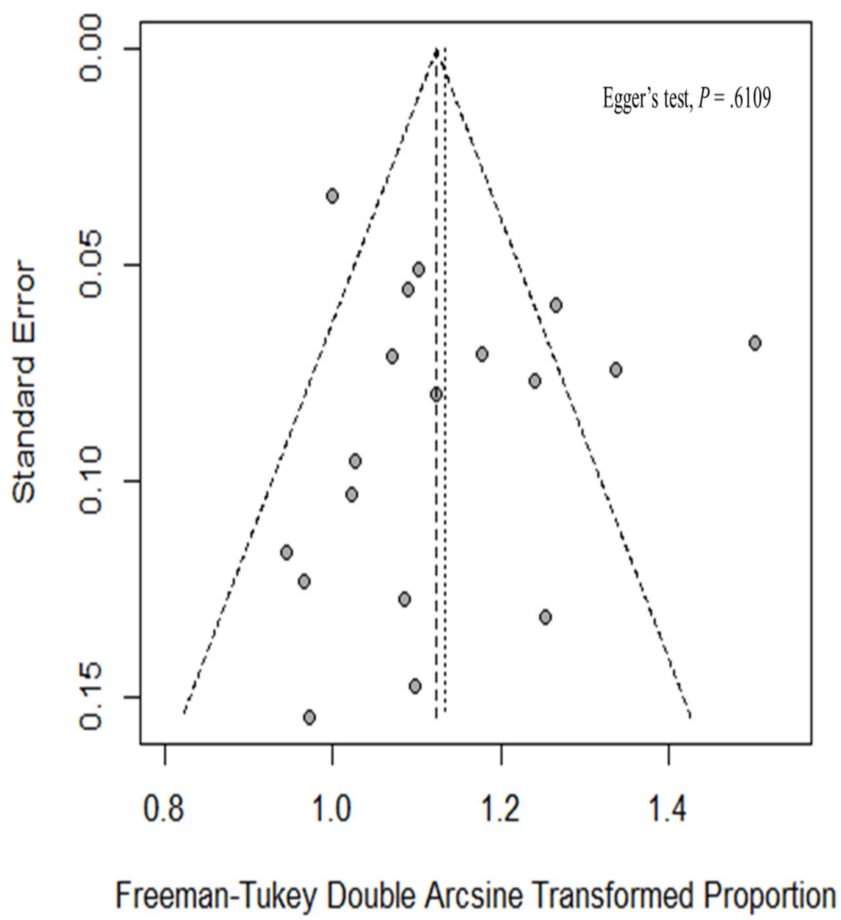

Funnel plot of 2-year local progression-free survival

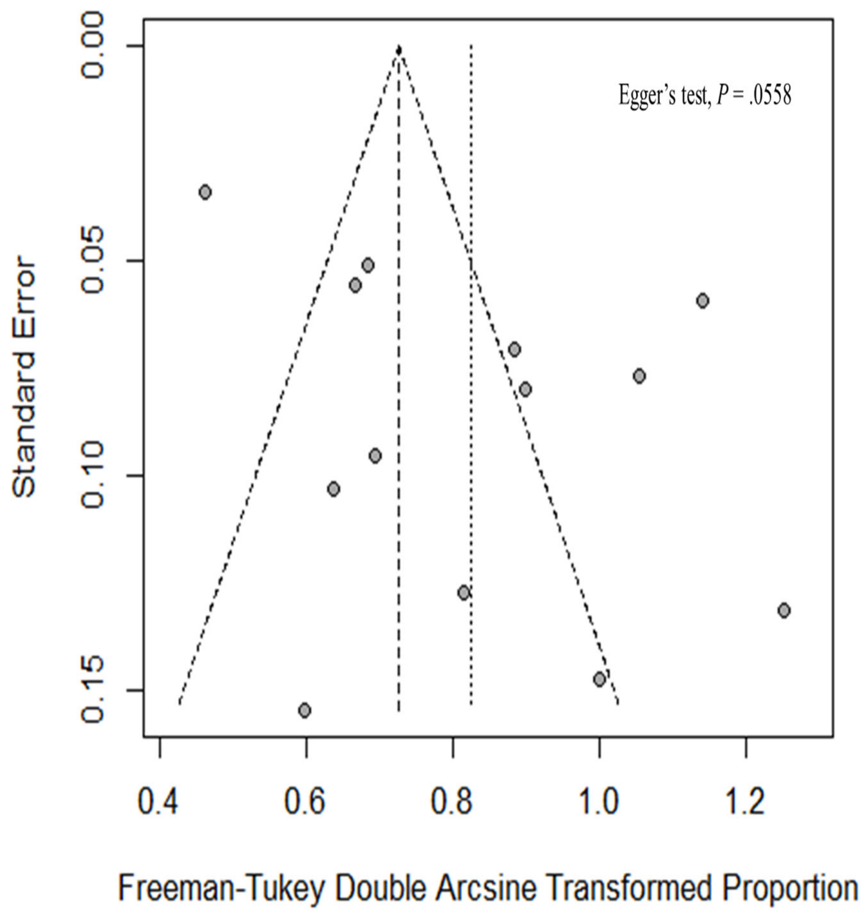

Funnel plot of 3-year local progression-free survival

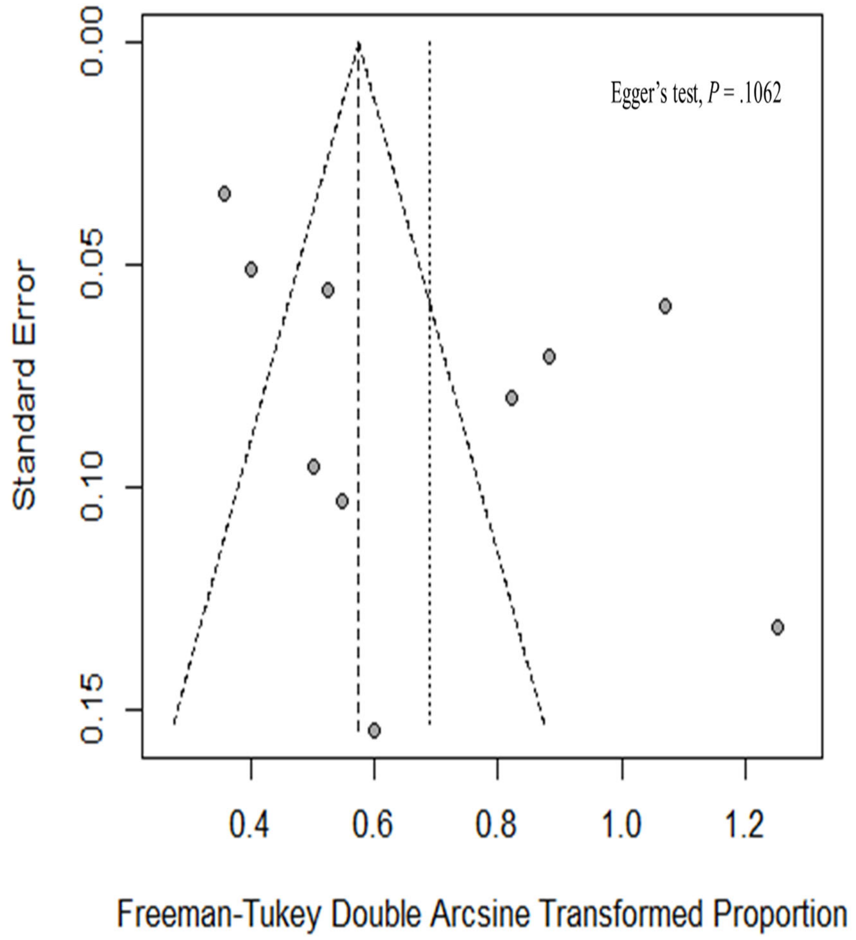

Funnel plot of 1-year progression-free survival

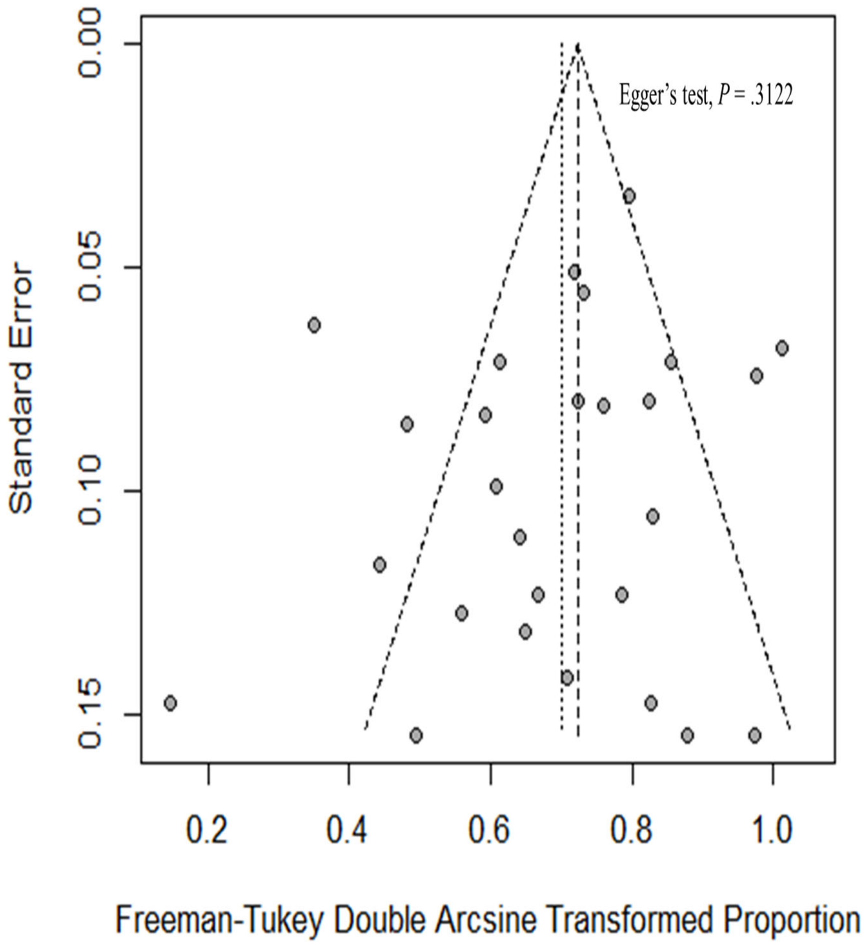

Funnel plot of 2-year progression-free survival

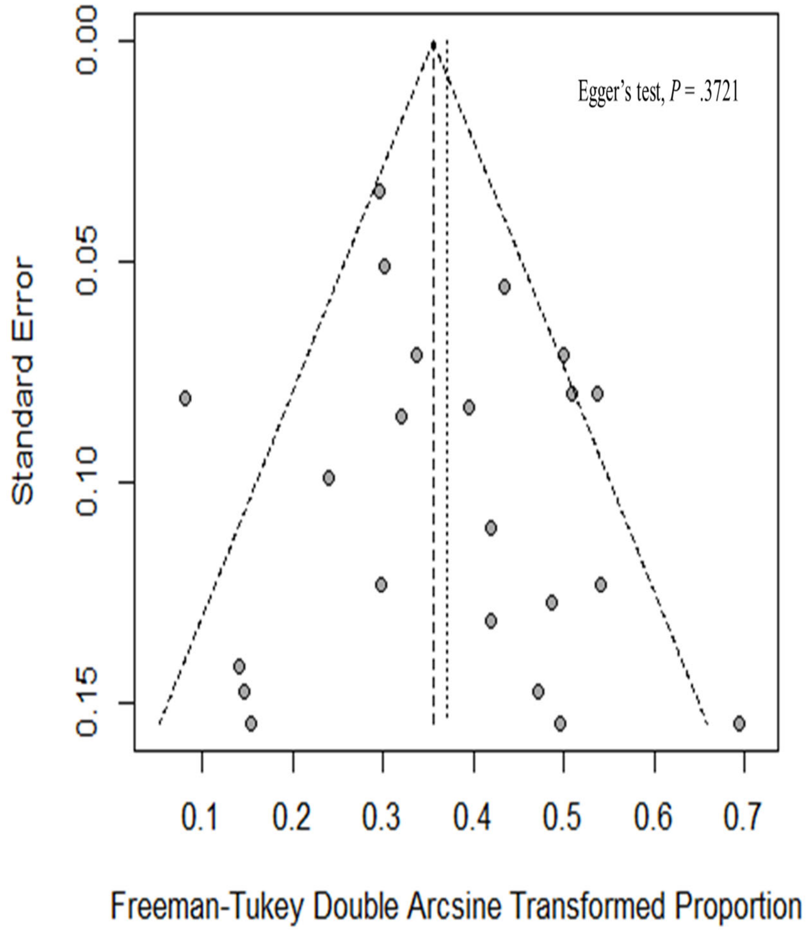

Funnel plot of 3-year progression-free survival

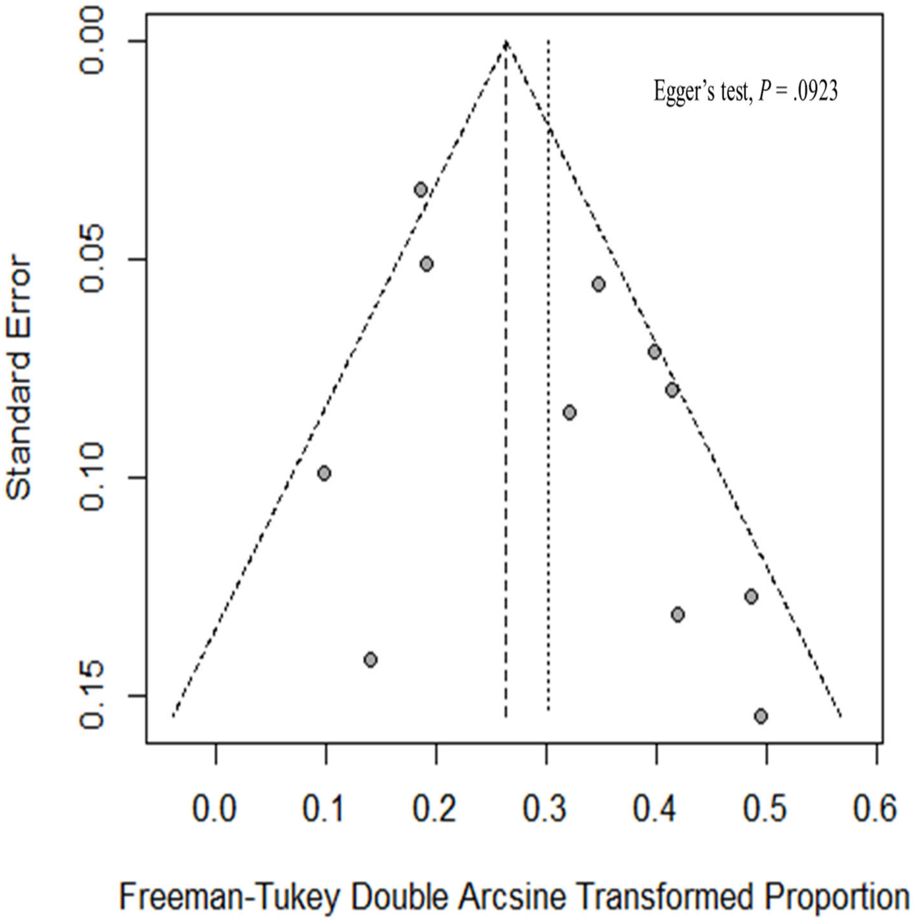

Funnel plot of 1-year overall survival

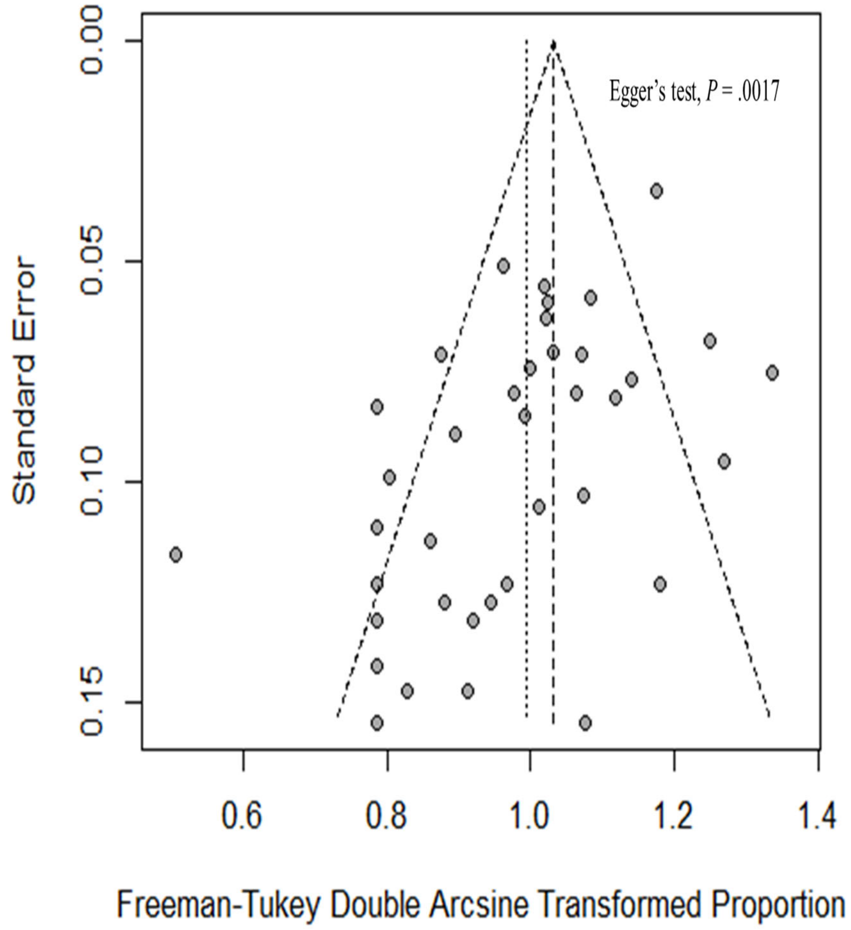

Funnel plot of 2-year overall survival

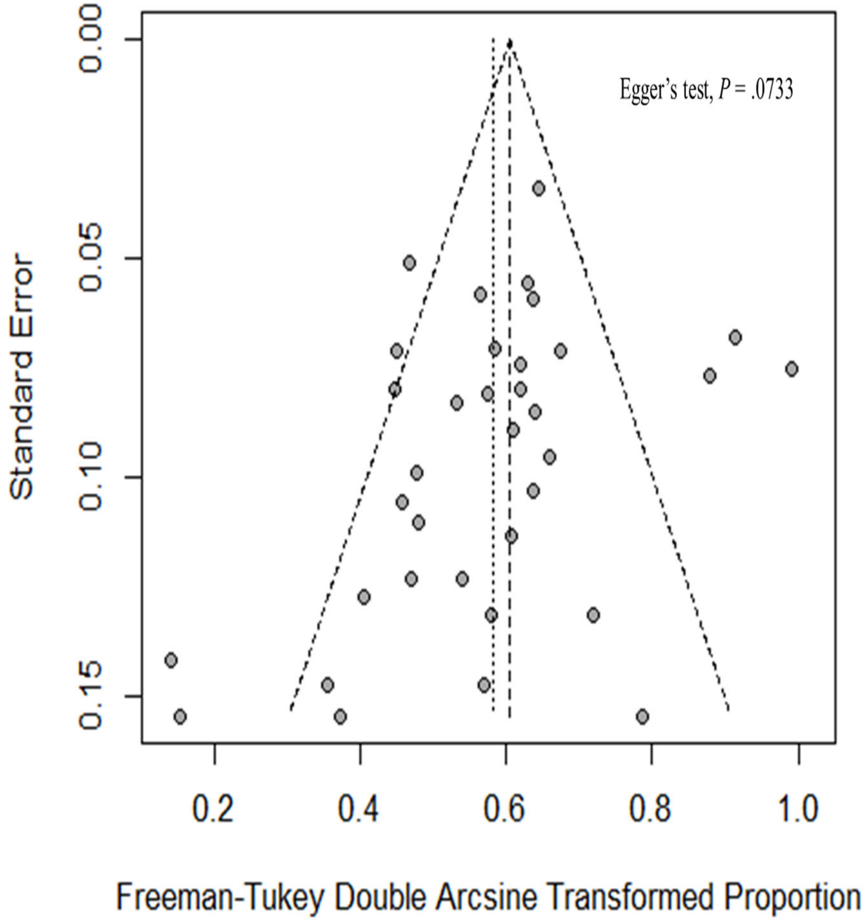

Funnel plot of 3-year overall survival

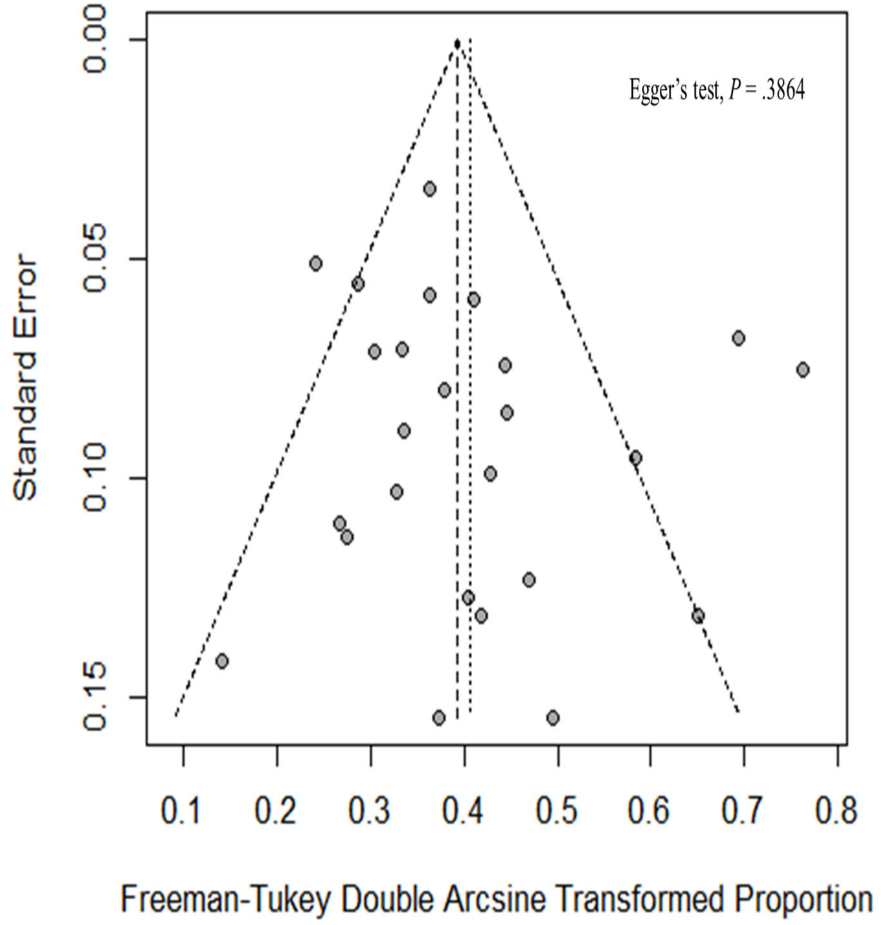

Supplementary Table 1. PRISMA 2020 Checklist

Section and Topic	Item #	Checklist item	Location where item is reported
**TITLE**			
Title	1	Identify the report as a systematic review.	Page 1, Lines 3-4
**ABSTRACT**			
Abstract	2	See the PRISMA 2020 for Abstracts checklist.	Page 1, Lines 39-41
**INTRODUCTION**			
Rationale	3	Describe the rationale for the review in the context of existing knowledge.	Page 2, Lines 74-91
Objectives	4	Provide an explicit statement of the objective(s) or question(s) the review addresses.	Page 2-3, Lines 92-98
**METHODS**			
Eligibility criteria	5	Specify the inclusion and exclusion criteria for the review and how studies were grouped for the syntheses.	Page 3, Lines 114-125
Information sources	6	Specify all databases, registers, websites, organisations, reference lists and other sources searched or consulted to identify studies. Specify the date when each source was last searched or consulted.	Page 3, Lines 101-102; 107-113
Search strategy	7	Present the full search strategies for all databases, registers and websites, including any filters and limits used.	Page 3, Lines 103-113, Supplementary Table 2
Selection process	8	Specify the methods used to decide whether a study met the inclusion criteria of the review, including how many reviewers screened each record and each report retrieved, whether they worked independently, and if applicable, details of automation tools used in the process.	Page 3, Lines 109-125
Data collection process	9	Specify the methods used to collect data from reports, including how many reviewers collected data from each report, whether they worked independently, any processes for obtaining or confirming data from study investigators, and if applicable, details of automation tools used in the process.	Page 3, Lines 127-128
Data items	10a	List and define all outcomes for which data were sought. Specify whether all results that were compatible with each outcome domain in each study were sought (e.g. for all measures, time points, analyses), and if not, the methods used to decide which results to collect.	Page 3, Lines 128-136
	10b	List and define all other variables for which data were sought (e.g. participant and intervention characteristics, funding sources). Describe any assumptions made about any missing or unclear information.	Page 3, Lines 126-136
Study risk of bias assessment	11	Specify the methods used to assess risk of bias in the included studies, including details of the tool(s) used, how many reviewers assessed each study and whether they worked independently, and if applicable, details of automation tools used in the process.	Page 3-4, Lines 137-142
Effect measures	12	Specify for each outcome the effect measure(s) (e.g. risk ratio, mean difference) used in the synthesis or presentation of results.	Page 3, Lines 128-136
Synthesis methods	13a	Describe the processes used to decide which studies were eligible for each synthesis (e.g. tabulating the study intervention characteristics and comparing against the planned groups for each synthesis (item #5)).	Page 4, Lines 144-149

Section and Topic	Item #	Checklist item	Location where item is reported
	13b	Describe any methods required to prepare the data for presentation or synthesis, such as handling of missing summary statistics, or data conversions.	All extracted data were used as reported in the original studies.
	13c	Describe any methods used to tabulate or visually display results of individual studies and syntheses.	Page 3, Lines 127-133
	13d	Describe any methods used to synthesize results and provide a rationale for the choice(s). If meta-analysis was performed, describe the model(s), method(s) to identify the presence and extent of statistical heterogeneity, and software package(s) used.	Page 4, Lines 143-156
	13e	Describe any methods used to explore possible causes of heterogeneity among study results (e.g. subgroup analysis, meta-regression).	Page 4, Lines 152-154
	13f	Describe any sensitivity analyses conducted to assess robustness of the synthesized results.	Not used
Reporting bias assessment	14	Describe any methods used to assess risk of bias due to missing results in a synthesis (arising from reporting biases).	Page 3-4, Lines 137-142
Certainty assessment	15	Describe any methods used to assess certainty (or confidence) in the body of evidence for an outcome.	Page 4, Lines 154
**RESULTS**			
Study selection	16a	Describe the results of the search and selection process, from the number of records identified in the search to the number of studies included in the review, ideally using a flow diagram.	Page 4, Lines 158-167; Figure 1
	16b	Cite studies that might appear to meet the inclusion criteria, but which were excluded, and explain why they were excluded.	Page 4, Lines 159-162
Study characteristics	17	Cite each included study and present its characteristics.	Page 4, Lines 168-186; Table 1
Risk of bias in studies	18	Present assessments of risk of bias for each included study.	Page 8-9, Lines 232-236; Supplementary Figure 1
Results of individual studies	19	For all outcomes, present, for each study: (a) summary statistics for each group (where appropriate) and (b) an effect estimate and its precision (e.g. confidence/credible interval), ideally using structured tables or plots.	Table 1-3, Figure 2-3, Supplementary Figure 1
Results of syntheses	20a	For each synthesis, briefly summarise the characteristics and risk of bias among contributing studies.	Page 6, Lines 195-202; Page 8, Lines 225-228
	20b	Present results of all statistical syntheses conducted. If meta-analysis was done, present for each the summary estimate and its precision (e.g. confidence/credible interval) and measures of statistical heterogeneity. If comparing groups, describe the direction of the	Page 6, Lines 203-209; Page 8, Lines

Section and Topic	Item #	Checklist item	Location where item is reported
		effect.	228-231
	20c	Present results of all investigations of possible causes of heterogeneity among study results.	Page 11, Lines 348-358
	20d	Present results of all sensitivity analyses conducted to assess the robustness of the synthesized results.	Not used
Reporting biases	21	Present assessments of risk of bias due to missing results (arising from reporting biases) for each synthesis assessed.	Page 8-9, Lines 232-236
Certainty of evidence	22	Present assessments of certainty (or confidence) in the body of evidence for each outcome assessed.	Page 4-8, Lines 169-231
**DISCUSSION**			
Discussion	23a	Provide a general interpretation of the results in the context of other evidence.	Page 9, Lines 238-254
	23b	Discuss any limitations of the evidence included in the review.	Page 11, Lines 358-367
	23c	Discuss any limitations of the review processes used.	Page 11, Lines 348-358
	23d	Discuss implications of the results for practice, policy, and future research.	Page 11, Lines 369-376
**OTHER INFORMATION**			
Registration and protocol	24a	Provide registration information for the review, including register name and registration number, or state that the review was not registered.	This systematic review was registered with the PROSPERO (CRD42024588555).
	24b	Indicate where the review protocol can be accessed, or state that a protocol was not prepared.	A protocol was not prepared
	24c	Describe and explain any amendments to information provided at registration or in the protocol.	Page 3, Lines 101-102
Support	25	Describe sources of financial or non-financial support for the review, and the role of the funders or sponsors in the review.	Page 15, Lines 398-402
Competing interests	26	Declare any competing interests of review authors.	Page 15, Line 407
Availability of data, code and other materials	27	Report which of the following are publicly available and where they can be found: template data collection forms; data extracted from included studies; data used for all analyses; analytic code; any other materials used in the review.	Table 1, 2

Supplementary Table 2. Search strategy and results

(1) PICO model

Population	Patients with liver-confined hepatocellular carcinoma
Intervention	Proton beam therapy
Comparison	Not specified
Outcomes	Survival and toxicity

(2) Keywords

PICO	Fields	Keywords	Remarks
P	MeSH	Pancreatic Neoplasms	A
	MeSH	Carcinoma, Pancreatic Ductal	
	MeSH	Pancreas	B
	TIAB	Pancrea*	
	TIAB	Neoplasm*	C
	TIAB	Tumor*	
	TIAB	Tumour*	
	TIAB	Carcinoma*	
	TIAB	Cancer*	
	TIAB	Malignan*	
		**B AND C**	**D**
	TIAB	Advance*	E
	TIAB	Locally advance*	
	TIAB	Unresectable	
	TIAB	Non-metastatic	
		**(A OR D) AND E**	**F**
I	MeSH	Radiotherapy, intensity-modulated	G
	MeSH	Radiosurgery	
	MeSH	Proton therapy	
	MeSH	Protons	
	MeSH	Radiotherapy	
	MeSH	Heavy Ion Radiotherapy	
	MeSH	Radiotherapy, High-Energy	
	TIAB	Intensity-modulated	
	TIAB	Volumetric-modulated	
	TIAB	Helical tomotherap*	
	TIAB	Targeted radiation	
	TIAB	IMRT	

	TIAB	Radiosurg*	
	TIAB	Stereotactic Radiation*	
	TIAB	Stereotactic Radiosurg*	
	TIAB	Stereotactic Radiotherap*	
	TIAB	Stereotactic ablative Radiation*	
	TIAB	Stereotactic ablative Radiosurg*	
	TIAB	Stereotactic ablative Radiotherap*	
	TIAB	SBRT	
	TIAB	SABR	
	TIAB	Particle beam	
	TIAB	Carbon beam	
	TIAB	Heavy Ion	
	TIAB	Carbon Ion	
	TIAB	Proton*	
	TIAB	Proton beam	
	TIAB	Radiotherap*	
	TIAB	Radiation therap*	
	TIAB	Radiation treatment*	
	TIAB	Targeted radiation	
	TIAB	External radiation	
	TIAB	External beam	
	TIAB	EBRT	
**O**	MeSH	Survival	**H**
	MeSH	Progression-free survival	
	MeSH	Disease-free survival	
	TIAB	Surviv*	
	TIAB	Progression free surviv*	
	TIAB	Event-free surviv*	
	TIAB	Disease free surviv*	
	TIAB	Overall surviv*	
	TIAB	Toxic*	
	TIAB	Toxin*	
	TIAB	Local Control*	
	**F AND G AND H**		

(3) Search strategy

DB	Search Strategy

**PubMed**	("Pancreatic Neoplasms"[MeSH Terms] OR "carcinoma, pancreatic ductal"[MeSH Terms] OR (("Pancreas"[MeSH Terms] OR "Pancreas"[Title/Abstract]) AND ("neoplasm*"[Title/Abstract] OR "tumor*"[Title/Abstract] OR "tumour*"[Title/Abstract] OR "carcinoma*"[Title/Abstract] OR "cancer*"[Title/Abstract] OR "malignan*"[Title/Abstract]))) AND ("advance*"[Title/Abstract] OR "locally advance*"[Title/Abstract] OR "Unresectable"[Title/Abstract] OR "Non-metastatic"[Title/Abstract]) AND ("radiotherapy, intensity modulated"[MeSH Terms] OR "Radiosurgery"[MeSH Terms] OR "Proton therapy"[MeSH Terms] OR "Protons"[MeSH Terms] OR "Radiotherapy"[MeSH Terms] OR "Heavy Ion Radiotherapy"[MeSH Terms] OR "radiotherapy, high energy"[MeSH Terms] OR ("Intensity-modulated"[Title/Abstract] OR "Volumetric-modulated"[Title/Abstract] OR "helical tomotherap*"[Title/Abstract] OR "Targeted radiation"[Title/Abstract] OR "IMRT"[Title/Abstract] OR "radiosurg*"[Title/Abstract] OR "stereotactic radiation*"[Title/Abstract] OR "stereotactic radiosurg*"[Title/Abstract] OR "stereotactic radiotherap*"[Title/Abstract] OR "stereotactic ablative radiation*"[Title/Abstract] OR "stereotactic ablative radiosurg*"[Title/Abstract] OR "stereotactic ablative radiotherap*"[Title/Abstract] OR "SBRT"[Title/Abstract] OR "SABR"[Title/Abstract] OR "Particle beam"[Title/Abstract] OR "Carbon beam"[Title/Abstract] OR "Heavy Ion"[Title/Abstract] OR "Carbon Ion"[Title/Abstract] OR "proton*"[Title/Abstract] OR "Proton beam"[Title/Abstract] OR "radiotherap*"[Title/Abstract] OR "radiation therap*"[Title/Abstract] OR "radiation treatment*"[Title/Abstract] OR "Targeted radiation"[Title/Abstract] OR "External radiation"[Title/Abstract] OR "External beam"[Title/Abstract] OR "EBRT"[Title/Abstract])) AND ("Survival"[MeSH Terms] OR "Progression-free survival"[MeSH Terms] OR "Disease-free survival"[MeSH Terms] OR ("surviv*"[Title/Abstract] OR "progression free surviv*"[Title/Abstract] OR "event free surviv*"[Title/Abstract] OR "disease free surviv*"[Title/Abstract] OR "overall surviv*"[Title/Abstract] OR "toxic*"[Title/Abstract] OR "toxin*"[Title/Abstract] OR "local control"[Title/Abstract]))
**EMBASE**	((('pancreas tumor'/exp OR 'pancreatic ductal carcinoma'/exp) OR (('pancreas'/exp OR 'pancrea*':ab,ti) AND ('Neoplasm*':ab,ti OR 'Tumor*':ab,ti OR 'Tumour*':ab,ti OR 'Carcinoma*':ab,ti OR 'Cancer*':ab,ti OR 'Malignan*':ab,ti))) AND ('Advance*':ab,ti OR 'Locally advance*':ab,ti OR 'Unresectable':ab,ti OR 'Non-metastatic':ab,ti)) AND (('intensity modulated radiation therapy'/exp OR 'radiosurgery'/exp OR 'proton therapy'/exp OR 'proton'/exp OR 'radiotherapy'/exp OR 'ion therapy'/exp OR 'megavoltage radiotherapy'/exp OR 'volumetric modulated arc therapy'/exp OR 'stereotactic radiosurgery'/exp OR 'stereotactic body radiation therapy'/exp OR 'particle therapy'/exp OR 'cancer radiotherapy'/exp OR 'tomotherapy'/exp OR 'external beam radiotherapy'/exp) OR ('Intensity-modulated':ab,ti OR 'Volumetric-modulated':ab,ti OR 'Helical tomotherap*':ab,ti OR 'Targeted radiation':ab,ti OR 'IMRT':ab,ti OR 'Radiosurg*':ab,ti OR 'Stereotactic Radiation*':ab,ti OR 'Stereotactic Radiosurg*':ab,ti OR 'Stereotactic Radiotherap*':ab,ti OR 'Stereotactic ablative Radiation*':ab,ti OR 'Stereotactic ablative Radiosurg*':ab,ti OR 'Stereotactic ablative Radiotherap*':ab,ti OR 'SBRT':ab,ti OR 'SABR':ab,ti OR 'Particle beam':ab,ti OR 'Carbon beam':ab,ti OR 'Heavy Ion':ab,ti OR 'Carbon Ion':ab,ti OR 'Proton*':ab,ti OR 'Proton beam':ab,ti OR 'Radiotherap*':ab,ti OR 'Radiation therap*':ab,ti OR 'Radiation treatment*':ab,ti OR 'Targeted radiation':ab,ti OR 'External radiation':ab,ti OR 'External beam':ab,ti OR

	'EBRT':ab,ti)) AND (('survival'/exp OR 'progression free survival'/exp OR 'disease free survival'/exp OR 'event free survival'/exp) OR ('Surviv*':ab,ti OR 'Progression free surviv*':ab,ti OR 'Event-free surviv*':ab,ti OR 'Disease free surviv*':ab,ti OR 'Overall surviv*':ab,ti OR 'Toxic*':ab,ti OR 'Toxin*':ab,ti OR 'Local Control*':ab,ti))
**Cochrane**	(((MeSH descriptor: [Pancreatic Neoplasms] explode all trees OR MeSH descriptor: [Carcinoma, Pancreatic Ductal] explode all trees) OR ((MeSH descriptor: [Pancreas] explode all trees OR (Pancreas):ti,ab,kw) AND ((neoplasm*):ti,ab,kw OR (tumor*):ti,ab,kw OR (tumour*):ti,ab,kw OR (carcinoma*):ti,ab,kw OR (cancer*):ti,ab,kw OR (malignan*):ti,ab,kw))) AND ((advance*):ti,ab,kw OR (locally NEXT advance*):ti,ab,kw OR (unresectable):ti,ab,kw OR ("non-metastatic"):ti,ab,kw)) AND ((MeSH descriptor: [Radiotherapy, Intensity-Modulated] explode all trees OR MeSH descriptor: [Radiosurgery] explode all trees OR MeSH descriptor: [Proton Therapy] explode all trees OR MeSH descriptor: [Protons] explode all trees OR MeSH descriptor: [Radiotherapy] explode all trees OR MeSH descriptor: [Heavy Ion Radiotherapy] explode all trees OR MeSH descriptor: [Radiotherapy, High-Energy] explode all trees) OR (("intensity-modulated"):ti,ab,kw OR ("volumetric-modulated"):ti,ab,kw OR (helical NEXT tomotherap*):ti,ab,kw OR ("targeted radiation"):ti,ab,kw OR (IMRT):ti,ab,kw OR (radiosurg*):ti,ab,kw OR (stereotactic NEXT radiation*):ti,ab,kw OR (stereotactic NEXT radiosurg*):ti,ab,kw OR (stereotactic NEXT radiotherap*):ti,ab,kw OR ("stereotactic ablative" NEXT radiation*):ti,ab,kw OR ("stereotactic ablative" NEXT radiosurg*):ti,ab,kw OR ("stereotactic ablative" NEXT radiotherap*):ti,ab,kw OR (SBRT):ti,ab,kw OR (SABR):ti,ab,kw OR ("particle beam"):ti,ab,kw OR ("carbon beam"):ti,ab,kw OR ("heavy ion"):ti,ab,kw OR ("carbon ion"):ti,ab,kw OR (proton*):ti,ab,kw OR ("proton beam"):ti,ab,kw OR (radiotherap*):ti,ab,kw OR (radiation NEXT therap*):ti,ab,kw OR (radiation NEXT treatment*):ti,ab,kw OR ("targeted radiation"):ti,ab,kw OR ("external radiation"):ti,ab,kw OR ("external beam"):ti,ab,kw OR (EBRT):ti,ab,kw)) AND ((MeSH descriptor: [Survival] explode all trees OR MeSH descriptor: [Progression-Free Survival] explode all trees OR MeSH descriptor: [Disease-Free Survival] explode all trees) OR ((surviv*):ti,ab,kw OR ("progression free" NEXT surviv*):ti,ab,kw OR ("event free" NEXT surviv*):ti,ab,kw OR ("disease free" NEXT surviv*):ti,ab,kw OR (overall NEXT surviv*):ti,ab,kw OR (toxic*):ti,ab,kw OR (toxin*):ti,ab,kw OR ("local control"):ti,ab,kw))
**Web of Science**	(((TI=("Pancrea*") OR AB=("Pancrea*")) AND (TI=("Neoplasm*") OR TI=("Tumor*") OR TI=("Tumour*") OR TI=("Carcinoma*") OR TI=("Cancer*") OR TI=("Malignan*") OR AB=("Neoplasm*") OR AB=("Tumor*") OR AB=("Tumour*") OR AB=("Carcinoma*") OR AB=("Cancer*") OR AB=("Malignan*"))) AND (TI=(Advance*) OR TI=("Locally advance*") OR TI=("Unresectable") OR TI=("Non-metastatic") OR AB=(Advance*) OR AB=("Locally advance*") OR AB=("Unresectable") OR AB=("Non-metastatic"))) AND (TI=("Intensity-modulated") OR TI=("Volumetric-modulated") OR TI=("Helical tomotherap*") OR TI=("Targeted radiation") OR TI=("IMRT") OR TI=("Radiosurg*") OR TI=("Stereotactic Radiation*") OR TI=("Stereotactic Radiosurg*") OR TI=("Stereotactic Radiotherap*") OR TI=("Stereotactic ablative Radiation*") OR TI=("Stereotactic ablative Radiosurg*") OR TI=("Stereotactic ablative Radiotherap*") OR TI=("SBRT") OR TI=("SABR") OR TI=("Particle beam") OR TI=("Carbon beam") OR TI=("Heavy Ion") OR TI=("Carbon Ion") OR TI=("Proton*") OR TI=("Proton beam") OR TI=("Radiotherap*") OR TI=("Radiation

	therap*") OR TI=("Radiation treatment*") OR TI=("Targeted radiation") OR TI=("External radiation") OR TI=("External beam") OR TI=("EBRT") OR TI=("ion therap*") OR TI=("particle therap*") OR AB=("Intensity-modulated") OR AB=("Volumetric-modulated") OR AB=("Helical tomotherap*") OR AB=("Targeted radiation") OR AB=("IMRT") OR AB=("Radiosurg*") OR AB=("Stereotactic Radiation*") OR AB=("Stereotactic Radiosurg*") OR AB=("Stereotactic Radiotherap*") OR AB=("Stereotactic ablative Radiation*") OR AB=("Stereotactic ablative Radiosurg*") OR AB=("Stereotactic ablative Radiotherap*") OR AB=("SBRT") OR AB=("SABR") OR AB=("Particle beam") OR AB=("Carbon beam") OR AB=("Heavy Ion") OR AB=("Carbon Ion") OR AB=("Proton*") OR AB=("Proton beam") OR AB=("Radiotherap*") OR AB=("Radiation therap*") OR AB=("Radiation treatment*") OR AB=("Targeted radiation") OR AB=("External radiation") OR AB=("External beam") OR AB=("EBRT") OR AB=("ion therap*") OR AB=("particle therap*")) AND (TI=(Surviv*") OR TI=("Progression free surviv*") OR TI=("Event-free surviv*") OR TI=("Disease free surviv*") OR TI=("Overall surviv*") OR TI=("Toxic*") OR TI=("Toxin*") OR TI=("Local Control*") OR AB=(Surviv*") OR AB=("Progression free surviv*") OR AB=("Event-free surviv*") OR AB=("Disease free surviv*") OR AB=("Overall surviv*") OR AB=("Toxic*") OR AB=("Toxin*") OR AB=("Local Control*"))

(4) Summary of search results

No.	DB	Results	Duplication
1	PubMed (Medline)	400	386
2	EMBASE	750	
3	Cochrane Library	33	
4	Web of Science	140	
Number of Search results (with duplication)		1,323	
**Number of Search results (without duplication)**		937	

Supplementary Table 3. Study details on chemotherapy

Author	RT	Study _date	ICT (%)	ICT_regimen (%)	Median duration of ICT (mo) (range)	CC RT (%)	CCRT regimen	Op after RT (%)
Passoni[22]	IMRT	2004-2019	100	Cap (1)/ Gem (11)/ PEXG (30)/ PDXG (6)/ PAXG (17)/ AG (29)/ FOLFININOX (3)/ GEMOX (2)/ Others (1)	6 (1-17)	93	Cap (100)	4
Ogawa_A[23]	IMRT	2016-2020	100	Gem (52)/ S-1 (9)/ m FOLFININOX (13)/ AG (26)		100	Gem (83)/ S-1 (17)	
Ogawa_B[23]	IMRT	2016-2020	100	Gem (50)/S-1 (14)/ AG (36)		100	Gem (86)/ S-1 (14)	
Argalacsova[24]	IMRT	2014-2021	100	m FOLFININOX (100)		0		21
Shi[25]	IMRT	2018-2021	100	AG (100)		0		
Roy[26]	IMRT	2012-2019	0		0	100	ABX (100)	0
Reyngold[27]	IMRT	2016-2019	98	FOLFININOX (57)/ AG (32)/ Others (11)	4 (0.5-13)	93	5-FU (96)/ Others (4)	
Felice[28]	IMRT	2015-2016	80	Gem-based (100)		0		
Lewis[29]	IMRT	2008-2011	0		0	100	Gem (100)	0
Cuneo[30]	IMRT	2014-2018	100	Gem-Adavosertib (100)	1.5	100	Gem-Adavosertib (100)	6
Oh[31]	IMRT	2011-2015	79	GemCDDP (89)/ Gem-erlo (5)/ GemCap (3)/ CAPOX (3)		100	Gem (79)/ Cap (21)	26
Goto[32]	IMRT	2001-2015	100	Gem (100)	1	100	Gem (96)/ S-1 (4)	
Park_A[33]	IMRT	2008-	100	Gem-based (53)/ FOLFININOX (45)/ FOLFOX	3.4 (0.2-	97	Gem (53)/ Cap (31)/	17

		2016		(2)	15.9)		5-FU (15)/ Flavopiridol (1)	
Colbert[34]	IMRT	2006-2016	100	5-FU-based / Gem-based/ Cet-based		100	5-FU-based (73)/ Gem-based (20)/ Cet-based (7)	9
Wang[35]	IMRT	2006-2013						
Jiang[36]	IMRT	2007-2012	0		0	100	Cap-erlo (100)	0
Chiorean[37]	IMRT	2006-2009	100	Gem-Sorafenib (100)	1	100	Gem-Sorafenib (100)	12
Ben-Josef[38]	IMRT	2006-2010	100	Gem (100)	0.7	100	Gem (100)	24
Abelson[39]	IMRT	2003-2008	28			100	Cap or 5-FU (100)	0
Milano[40]	IMRT	1998-2003				100	5-FU (100)	0
Van ’t Land[41]	SBRT	2019-2021	100	FOLFININOX (100)	2	100	IMM-101 (100)	21
Reyngold[42]	SBRT	2016-2019	100	FOLFININOX (63)/ AG (25)/ Others (12)	4.5 (1.6-10.9)	0		17
Hurmuz[43]	SBRT	2009-2022	67	Gem (50)/ FOLFININOX (50)		34	Cap (100)	0
Doppenberg[44]	SBRT	2016-2022	100	FOLFININOX (88)/Gem-based (12)		0		
Comito[45]	SBRT	2011-2021	54	Gem (9)/ FOLFININOX (24)/AG (22)/ GEMOX (28)/ PEFG (13)/ Others (4)		0		3
Lee[46]	SBRT	2017-2021	100			0		
Kaucic[47]	SBRT	2017-2021				0		
Kaucic[48]	SBRT	2017-				0		

		2020						
Zhu[49]	SBRT	2016-2018	0		0	0		
Teriaca[50]	SBRT	2014-2017	100	FOLFININOX (100)	4 (1-4)	0		18
Qing[51]	SBRT	2016-2018	0		0	100	S-1 (100)	0
Bouchart[52]	SBRT	2017-2020	100	mFOLFININOX or AG (100)	3.4 (IQR 2.5-4.0)	0		38
Jung[53]	SBRT	2011-2016	14	Gem-based (77)/ mFOLFININOX (23)		0		7
Quan[54]	SBRT	2011-2013	100	GemCap (100)	3	0		13
Jumeau[55]	SBRT	2010-2016	29			0		0
Heerkens[56]	SBRT	2013-2016	0		0	0		0
Park_B[33]	SBRT	2008-2016	95	Gem-based (50)/ FOLFININOX (45)/ FOLFOX (5)	4.1 (0.5-11.7)	0		7
Mellon[57]	SBRT	2009-2014	100	GTX (10)/ FOLFININOX (43)/ AG (12)/ Gem (35)		0		10
Herman[58]	SBRT	2010-2012	90	Gem (100)	0.7	0		8
Gurka[59]	SBRT	2009-2011	100	Gem (100)	0.7	0		0
Schellenberg[60]	SBRT	2006-2007	100	Gem (100)	0.7 (0.2-1.2)	0		
Mahadevan[61]	SBRT	2007-2010	100	Gem (100)	1.5	0		
Shen[62]	SBRT	2009-2010				0		
Mahadevan[63]	SBRT	2005-	0		0	0		

		2007						
Schellenberg[64]	SBRT	2004-2006	100	Gem (100)	0.7 (0.2-0.7)	0		0
Okamoto[65]	PBT	2016-2020	84	AG (78)/ FOLFININOX (13)/ Gem (3)/ GemTGO (3)/ TGO(3)	2.9 (1.0-11.4)	100	Gem (59)/ TGO (41)	
Lautenschlaeger[66]	PBT	2017-2021	73					13
Ami[67]	PBT	2009-2021	53	FOLFININOX (6)/ AG (15)/ FOLFININOX -AG (48)/Others (31)		93	Gem (85)/ S-1 (15)	
Yu[68]	PBT	2015-2016	90	Gem-based (89)/ S-1 (11)		60	Cap (100)	
Kim[69]	PBT	2013-2019	24	FOLFININOX (47)/ AG (21)/ Gem-erlo (16)/ GemCap (11)/ GemCDDP (5)	6.4 (1.8-18.0)	90	Cap (97)/ 5-FU (3)	5
Hiroshima[70]	PBT	2009-2016	76	Gem / S-1/ FOLFOX/ nab-PTX/ Others		100	Gem (90)/ S-1 (10)	5
Kawashiro[71]	PBT	2012-2014	74	Gem (49)/ Gem+S-1(30)/ S-1 (9)/ FOLFININOX (6)/ Others (6)	2 (1-10)	78	Gem (87)/ S-1 (11)/ Gem+S-1 (2)	
Maemura[72]	PBT	2010-2015	100	Gem+S-1(100)		100	S-1	
Shinoto[73]	PBT	2007-2012	0		0	99	Gem (100)	7
Sachsmann[74]	PBT	2010-NR	73	Gem (63)/ FOLFININOX (37)		100	Cap (100)	27

Abbreviations: RT – radiotherapy; ICT – induction chemotherapy before RT; CCRT – concurrent chemoradiotherapy; Op – operation; IMRT – intensity-modulated radiotherapy; SBRT – stereotactic body radiotherapy; PBT – particle beam therapy; Cap – capecitabine; Gem – gemcitabine; PEXG – cisplatin-epirubicin-capecitabine-gemcitabine; PDXG – cisplatin-docetaxel-capecitabine- gemcitabine; PAXG – cisplatin-nab-paclitaxel-capecitabine-gemcitabine; AG – nab-paclitaxel-gemcitabine; FOLFININOX – fluorouracil-lecovorin-irinotecan-oxaliplatin; GEMOX – gemcitabine-oxaliplatin; ABX – nab-paclitaxel; 5-FU, 5-fluorouracil; CDDP – cisplatin; erlo – erlotinib; FOLFOX – 5-fluorouracil-l-leucovorin-oxaliplatin; Cet – Cetuximab; CAPOX – capecitabine- oxaliplatin; PEFG – cisplatin-epirubicin-5-fluorouracil-gemcitabine; FOLFIRI – 5-fluorouracil- l-leucovorin- irinotecan; GTX – gemcitabine-docetaxel-capecitabine; GemTGO – gemcitabine-tegafur-gimeracil-oteracil potassium; CapOX – capecitabine-oxaliplatin; nab-PTX – nanoparticle albumin-bound paclitaxel.

Supplementary Table 4. Study details on radiotherapy (RT)

Author	RT	Tumor location (HN vs. BT) (%)	LN (+) (%)	Median tumor size (cm) (range)	Median PTV (mL) (range)	Median RT dose (Gy)	Median No. of fraction (range)	mBED (Gy_{10})	RT machine
Passoni[22]	IMRT	67/33				44.3 (40-58)	15	57.3	Tomotherapy
Ogawa_A[23]	IMRT	39/61			213 (146-483)	48	15	63.4	TrueBeam STx
Ogawa_B[23]	IMRT	71/29			193 (129-452)	48	15	63.4	TrueBeam STx
Argalacsova[24]	IMRT					39.9	15	50.5	Tomotherapy
Shi[25]	IMRT	50/50				SIB: 50/30	10	39.0	Tomotherapy
Roy[26]	IMRT	71/29		4.4 (2.0-6.5)		50.4	28	59.5	
Reyngold[27]	IMRT	62/38	49	3.8 (1.4-7.4)		75 (67.5 or 75)	25 (15 or 25)	97.5	Varian linear accelerator
Felice[28]	IMRT	70/30	40		97 (56-269)	52	13	72.8	
Lewis[29]	IMRT		50	3.5 (3.3-4)		SIB: 57/45	25	53.1	Tomotherapy
Cuneo[30]	IMRT		15	3 (1.4-7)		52.5	25	63.5	
Oh[31]	IMRT	55/45	53	3.8 (1.5-7.2)		SIB: 55/44	22	52.8	
Goto[32]	IMRT	59/37		3 (1-7)		48 (39-51)	15	63.4	
Park_A[33]	IMRT			3.4 (1.2-8.9)		NR (45-56)	NR (25-28)		
Colbert[34]	IMRT	52/48	27	3.7 (2.18)		63 (63-70)	28 (15-28)	77.2	
Wang[35]	IMRT					46 (26.8-54)	23	54.3	
Jiang[36]	IMRT					50.4	28	59.5	
Chiorean[37]	IMRT	78/22	37			SIB: 50/45	25	53.1	
Ben-Josef[38]	IMRT	86/14		3.3 (1.6-7.1)		55 (50-60)	25 (24-25)	67.1	
Abelson[39]	IMRT	78/22	39			54 (39.6-59.4)	30 (22-33)	63.7	Dual energy or Trilogy
Milano[40]	IMRT		36			59.4 (50.4-59.4)	33 (28-33)	70.1	
Van 't Land[41]	SBRT	71/29		3.1 (2.5-4)		40	5	72.0	Cybernife
Reyngold[42]	SBRT	50/50	71	3.8 (1.1-9.0)		30 (27-33)	3	60.0	

Hurmuz[43]	SBRT			3.5 (2.7-4)		35 (33-50)	5 (3 or 5)	59.5	Synergy, Novalis, or Cybernife
Doppenberg[44]	SBRT	68/32				40 (32-40)	5 (4 or 5)	72.0	MRgRT
Comito[45]	SBRT	84/16	0	3.7 (1.4-9.3)	71 (18-321)	45	6	78.8	RapidArc
Lee[46]	SBRT						5		MRIdian, TrueBeam-STX
Kaucic[47]	SBRT	76/24			56 (10-162)	45 (32 or 45)	3 (1 or 3 or 5)	112.5	Varian EDGE linear accelerator
Kaucic[48]	SBRT	73/27			104 (33-218)	40 (36 or 40 or 45)	5 (3 or 5)	72.0	Varian EDGE linear accelerator
Zhu[49]	SBRT	67/33	43	3.5 (2.9-4.3)		36 (35-40)	5	61.2	Cyberknife
Teriaca[50]	SBRT	59/41		3.9 (2-7)		40	5	72.0	
Qing[51]	SBRT	100/0	31	3.7 (2.3-5)	27 (12-48)	40 (35-45)	5	72.0	Cyberknife
Bouchart[52]	SBRT						5		Elekta InfinityTM linear accelerator equipped with Agility^{TM}
Jung[53]	SBRT	55/45	24			28 (24-36)	4 (4-5)	47.6	TrueBeam STx
Quan[54]	SBRT					36	3	79.2	
Jumeau[55]	SBRT	65/35				30 (30-35)	5 (5-6)	48.0	Cyberknife
Heerkens[56]	SBRT	85/15			82 (15-198)	24	3	43.2	Elekta Synergy
Park_B[33]	SBRT			3.2 (1.2-6.1)		NR (30-33)	5		
Mellon[57]	SBRT	69/31	59			SIB: 40/30 (28-50)	5	48.0	Varian Truebeam or Trilogy
Herman[58]	SBRT	84/16			71 (31-225)	33	5	54.8	TRueBeam or Others
Gurka[59]	SBRT	70/30	60		360 (154-548)	25	5	37.5	Cyberknife
Schellenberg[60]	SBRT		20		41 (12-84)	25	1	87.5	Varian trilogy

									21EX
Mahadevan[61]	SBRT	90/10				24 (24 or 30)	3	43.2	Cyberknife
Shen[62]	SBRT	65/35			47 (26-64)	45 (32-55)	4 (3-6)	95.6	Cyberknife
Mahadevan[63]	SBRT	56/44				30 (24-36)	3	60.0	Cyberknife
Schellenberg[64]	SBRT	88/12			48 (22-84)	25	1	87.5	Cyberknife
Okamoto[65]	PBT	39/61	18			55.2	12	80.6	C-ion beams
Lautenschlaeger[66]	PBT								Proton beam
Ami[67]	PBT	0/100	31	3.6 (1.5-7)		67.5	25	85.7	Proton beam
Yu[68]	PBT	50/50	20			65.4 (62.4-68.4)	33 (32-34)	79.0	Proton beam followed by carbon boost
Kim[69]	PBT	61/39	26	3.7 (2.2-7.3)		SIB: 45 (45-50) /30	10	39.0	Proton beam
Hiroshima[70]	PBT		36			60 (50-67.5)	25 (25-33)	72.0	Proton beam
Kawashiro[71]	PBT	42/58		3.5 (2.1-11.0)	178 (40-412)	55.2 (52.8 or 55.2)	12	80.6	C-ion beams
Maemura[72]	PBT	80/20		2.9+/-0.9		50	25	60.0	Proton beam
Shinoto[73]	PBT	53/47				NR (43.2-55.2)	12		C-ion beams
Sachsmann[74]	PBT		27			59.4	33	70.1	Proton beam

Abbreviations: HN – head or neck; BT – body or tail; LN = lymph node; PTV – planning target volume; No. – number; mBED – median biologically equivalent dose, which was calculated using an α/β ratio of 10; IMRT – intensity-modulated radiotherapy; SBRT – stereotactic body radiotherapy; PBT – particle beam therapy; SIB – simultaneous integral boost; NR – not reported.

Supplementary Table 5. Pooled rates of local progression-free survival (LPFS) and progression-free survival (PFS)

Group	Cohorts	N	P, Heterogeneity	I^2	Random Event rate (95% CI)	P (between groups)
1-year LPFS	19	926	<0.0001	74.70%	0.83 (0.78-0.88)	
IMRT	6	349	0.1317	41.03%	0.77 (0.69-0.84)	0.1900
PBT	5	215	0.1150	46.13%	0.86 (0.78-0.93)	
SBRT	8	362	<0.0001	81.72%	0.86 (0.75-0.94)	
2-year LPFS	13	695	<0.0001	92.54%	0.54 (0.39-0.69)	
IMRT	5	331	<0.0001	93.15%	0.49 (0.24-0.74)	0.6911
PBT	5	215	<0.0001	90.54%	0.62 (0.38-0.84)	
SBRT	3	149	0.0693	62.54%	0.50 (0.35-0.65)	
3-year LPFS	10	627	<0.0001	95.04%	0.40 (0.22-0.59)	
IMRT	5	331	<0.0001	95.06%	0.41 (0.14-0.71)	0.9022
PBT	3	162	<0.0001	95.75%	0.45 (0.09-0.85)	
SBRT	2	134	<0.0001	95.02%	0.32 (0.03-0.73)	
1-year PFS	28	1059	<0.0001	77.87%	0.41 (0.34-0.48)	
IMRT	10	378	<0.0001	77.80%	0.33 (0.21-0.47)	0.2168
PBT	4	112	0.6540	0	0.48 (0.38-0.58)	
SBRT	14	569	<0.0001	83.46%	0.43 (0.33-0.54)	
2-year PFS	22	847	0.0005	57.31%	0.11 (0.08-0.16)	
IMRT	7	328	0.4510	0%	0.06 (0.04-0.10)	0.0058
PBT	4	112	0.4670	0%	0.18 (0.11-0.27)	
SBRT	11	407	0.0008	66.95%	0.12 (0.06-0.19)	
3-year PFS	11	591	0.0024	63.34%	0.07 (0.03-0.12)	
IMRT	6	317	0.0542	53.96%	0.04 (0.00-0.11)	0.3617
PBT	2	91	0.3674	0%	0.11 (0.05-0.19)	
SBRT	3	183	0.0148	76.26%	0.10 (0.02-0.21)	

Abbreviations: N – number of patients; IMRT – intensity-modulated radiotherapy; SBRT – stereotactic body radiotherapy; PBT – particle beam therapy.

Supplementary Table 6. Severe toxicities ≥grade 3

Author	RT	Toxicity criteria	Acute hematologic toxicities	Acute gastrointestinal toxicities	Late gastrointestinal toxicities
Passoni[22]	IMRT	CTCAE v5.0	4	1	4
Ogawa_A[23]	IMRT	CTCAE v5.0			4
Ogawa_B[23]	IMRT	CTCAE v5.0			43
Argalacsova[24]	IMRT				
Shi[25]	IMRT	CTCAE v4.03	18	0	
Roy[26]	IMRT	CTCAE v4.0	14	14	0
Reyngold[27]	IMRT	CTCAE v4.0			10
Felice[28]	IMRT	CTCAE v4.0	0	0	0
Lewis[29]	IMRT	CTCAE v3.0	0	0	0
Cuneo[30]	IMRT	CTCAE v3.0	18	9	
Oh[31]	IMRT	CTCAE v4.03	13	0	0
Goto[32]	IMRT	CTCAE v4.0		0	4
Park_A[33]	IMRT	CTCAE v4.0	26	0	2
Colbert[34]	IMRT	CTCAE v4.0		0	10
Wang[35]	IMRT				
Jiang[36]	IMRT	CTCAE v3.0	73	0	
Chiorean[37]	IMRT	CTCAE v3.0	44	0	50
Ben-Josef[38]	IMRT		78	8	
Abelson[39]	IMRT	CTCAE v3.0		6	0
Milano[40]	IMRT	RTOG			0
Van ’t Land[41]	SBRT			3	0
Reyngold[42]	SBRT	CTCAE v4.0		0	0
Hurmuz[43]	SBRT	CTCAE v4.0	0	0	8
Doppenberg[44]	SBRT	CTCAE v5.0		0	3
Comito[45]	SBRT	CTCAE v5.0		0	0
Lee[46]	SBRT	CTCAE v5.0		0	
Kaucic[47]	SBRT	CTCAE v4.03		0	0
Kaucic[48]	SBRT	CTCAE v4.03		0	0
Zhu[49]	SBRT	CTCAE v4.0		0	2
Teriaca[50]	SBRT	CTCAE v4.0		5	3
Qing[51]	SBRT	CTCAE v4.0	19	0	0
Bouchart[52]	SBRT	CTCAE v4.0			0
Jung[53]	SBRT	CTVAE v4.03		0	3
Quan[54]	SBRT	CTCAE v4.0		0	0
Jumeau[55]	SBRT	CTCAE v4.0		0	6
Heerkens[56]	SBRT			0	0
Park_B[33]	SBRT	CTCAE v4.0	5	0	0
Mellon[57]	SBRT				
Herman[58]	SBRT	CTCAE v4.0	12	2	9
Gurka[59]	SBRT	CTCAE v3.0	0	0	0
Schellenberg[60]	SBRT	CTCAE v3.0		0	5
Mahadevan[61]	SBRT	CTCAE v3		0	8
Shen[62]	SBRT	RTOG	0	0	
Mahadevan[63]	SBRT			0	6

Schellenberg[64]	SBRT	CTCAE v3.0		6	13
Okamoto[65]	PBT	CTCAE v4.0	36	0	2
Lautenschlaeger[66]	PBT				
Ami[67]	PBT	CTCAE v5.0	36	8	8
Yu[68]	PBT	CTCAE v4.03	10	0	0
Kim[69]	PBT	CTCAE v4.03	0	0	0
Hiroshima[70]	PBT	CTCAE v4.0	45	0	0
Kawashiro[71]	PBT	CTCAE v4.0	26	0	1
Maemura[72]	PBT	CTCAE v4.0	0	10	
Shinoto[73]	PBT	CTCAE v3.0	53	0	1
Sachsmann[74]	PBT		0	0	0

Abbreviations: RT – radiotherapy; IMRT – intensity-modulated radiotherapy; SBRT – stereotactic body radiotherapy; PBT – particle beam therapy; CTCAE – Common Terminology Criteria for Adverse Events; RTOG – Radiation Therapy Oncology Group.
